# Yellow Fever Virus Maintained by *Sabethes* Mosquitoes during the Dry Season in Cerrado, a Semiarid Region of Brazil, in 2021

**DOI:** 10.3390/v15030757

**Published:** 2023-03-15

**Authors:** Cirilo H. de Oliveira, Miguel S. Andrade, Fabrício S. Campos, Jader da C. Cardoso, Maria Eduarda Gonçalves-dos-Santos, Ramon Silva Oliveira, Sandy Micaele Aquino-Teixeira, Aline AS Campos, Marco AB Almeida, Danilo Simonini-Teixeira, Anaiá da P. Sevá, Andrea Oliveira Dias Temponi, Fernando Maria Magalhães, Agna Soares da Silva Menezes, Bartolomeu Teixeira Lopes, Hermes P. Almeida, Ana Lúcia Pedroso, Giovani Pontel Gonçalves, Danielle Costa Capistrano Chaves, Givaldo Gomes de Menezes, Sofía Bernal-Valle, Nicolas FD Müller, Luis Janssen, Edmilson dos Santos, Maria A. Mares-Guia, George R. Albuquerque, Alessandro PM Romano, Ana C. Franco, Bergmann M. Ribeiro, Paulo M. Roehe, Ricardo Lourenço-de-Oliveira, Filipe Vieira Santos de Abreu

**Affiliations:** 1 Insect Behavior Laboratory, Federal Institute of Northern Minas Gerais, Salinas 39560-000, MG, Brazil; 2 Baculovirus Laboratory, Department of Cell Biology, Institute of Biological Sciences, University of Brasilia, Brasília 70910-900, DF, Brazil; 3 Department of Molecular Biology, Sabin Diagnóstico e Saúde, Brasília 70632-340, DF, Brazil; 4 Bioinformatics and Biotechnology Laboratory, Campus of Gurupi, Federal University of Tocantins, Gurupi 77410-570, TO, Brazil; 5 Institute of Basic Health Sciences, Federal University of Rio Grande do Sul, Porto Alegre 90035-003, RS, Brazil; 6 State Center of Health Surveillance, Rio Grande do Sul State Health Department, Porto Alegre 90610-000, RS, Brazil; 7 Pan American Health Organization, World Health Organization Office in Brazil, Brasília 70800-400, DF, Brazil; 8 Department of Agricultural and Environmental Sciences, Santa Cruz State University, Ilhéus 45662-900, BA, Brazil; 9 Health Department of the State of Minas Gerais, State Coordination for Arbovirus Surveillance, Belo Horizonte 31630-901, MG, Brazil; 10 Flavivirus Laboratory, Instituto Oswaldo Cruz, Fiocruz, Rio de Janeiro 21040-360, RJ, Brazil; 11 General Coordination of Arbovirus Surveillance, Ministry of Health, Brasília 70058-900, DF, Brazil; 12 Laboratório de Mosquitos Transmissores de Hematozoários, Instituto Oswaldo Cruz, Fiocruz, Rio de Janeiro 21040-360, RJ, Brazil

**Keywords:** Arbovirus, mosquito vector, Sabethini, Cerrado, genomic surveillance, MinION

## Abstract

In recent decades, waves of yellow fever virus (YFV) from the Amazon Rainforest have spread and caused outbreaks in other regions of Brazil, including the Cerrado, a savannah-like biome through which YFV usually moves before arriving at the Atlantic Forest. To identify the vectors involved in the maintenance of the virus in semiarid environments, an entomological survey was conducted after confirmation of yellow fever (YF) epizootics at the peak of the dry season in the Cerrado areas of the state of Minas Gerais. In total, 917 mosquitoes from 13 taxa were collected and tested for the presence of YFV. Interestingly, mosquitoes of the *Sabethes* genus represented 95% of the diurnal captured specimens, displaying a peak of biting activity never previously recorded, between 4:30 and 5:30 p.m. Molecular analysis identified three YFV-positive pools, two from *Sabethes chloropterus*—from which near-complete genomes were generated—and one from *Sa. albiprivus*, whose low viral load prevented sequencing. *Sa. chloropterus* was considered the primary vector due to the high number of copies of YFV RNA and the high relative abundance detected. Its bionomic characteristics allow its survival in dry places and dry time periods. For the first time in Brazil, *Sa. albiprivus* was found to be naturally infected with YFV and may have played a role as a secondary vector. Despite its high relative abundance, fewer copies of viral RNA were found, as well as a lower Minimum Infection Rate (MIR). Genomic and phylogeographic analysis showed that the virus clustered in the sub-lineage YFV_PA-MG_, which circulated in Pará in 2017 and then spread into other regions of the country. The results reported here contribute to the understanding of the epidemiology and mechanisms of YFV dispersion and maintenance, especially in adverse weather conditions. The intense viral circulation, even outside the seasonal period, increases the importance of surveillance and YFV vaccination to protect human populations in affected areas.

## 1. Introduction

The yellow fever virus (YFV)—family *Flaviviridae*, genus *Flavivirus*—is the causative agent of yellow fever (YF), an endemic disease in tropical regions of Africa and the Americas [1]. In humans, YF has a wide spectrum of severity, from subclinical presentations to severe manifestations and, sometimes, fatal outcomes. There is no specific treatment for YF. Vaccination is the main direct prophylactic method and is highly recommended in risk areas by the Brazilian Governmental Health Agencies [2,3].

In the Americas, YFV is maintained in a sylvatic cycle between non-human primates (NHPs) and sylvatic mosquitoes, mainly those of the *Haemagogus* and *Sabethes* genera [4,5,6,7]. In this situation, humans can be infected after being bitten by mosquitoes near to or within forested areas [2,7]. The rainforest of the Amazon region is considered an endemic area for YFV, as virus circulation is perennial. However, expansion waves of sylvatic YF have been reported outside the Amazon and have reached other biomes, such as the Cerrado (a savannah-like biome) and the Atlantic Forest (a tropical rainforest biome) [6,7]. Accordingly, when spreading out from the endemic Amazon region, YFV usually reaches the Cerrado in the states of Goiás (GO) and Minas Gerais (MG). Depending on several factors (not entirely known), including weather conditions and the presence of susceptible hosts, the virus can continue to spread to areas covered by the Atlantic Forest in the Southeast Region of Brazil [7,8,9,10]. Since the early 2000s, the invasion of these biomes has caused outbreaks generating great public health concerns [7].

Between 2014 and 2021, Brazil witnessed the largest YF epidemic of the last 80 years, which generated 2267 human cases and thousands of epizootics [7,9,11,12,13,14,15,16,17,18]. It has been demonstrated that, after spilling out from the Amazon region and crossing the Cerrado areas between 2014 and 2016, YFV reached the Atlantic Forest of the Southeast Region, in which the highest human population density in the country is concentrated. This area had been considered free of YFV since the 1940s, but was severely affected by this outbreak, mainly between 2016 and 2018, with 752 human deaths attributed to YF [16,17,19]. From 2018 to 2021, the virus kept on spreading across the Atlantic Forest, eventually reaching the South Region and the meridional limit of its distribution [15]. Genomic analyses showed that the virus that circulated from 2014 to 2021 belongs to the South American 1E genotype, divided into two sub-lineages from the Cerrado regions of GO. The first sub-lineage, named YFV_MG/ES/RJ/BA_, crossed the state of MG from west to east and reached the states of Espírito Santo (ES), Rio de Janeiro (RJ) and Bahia (BA); the second sub-lineage, named YFV_MG/SP/RS_, passed through west MG, invaded the state of São Paulo (SP) and then entered the states of Paraná (PR), Santa Catarina (SC) and Rio Grande do Sul (RS) [12,15,20,21,22,23]. Entomological investigations carried out during the 2014–2021 YFV wave pointed to the mosquitoes *Haemagogus janthinomys* and *Hg. leucocelaenus* as primary vectors of YFV in southeastern Atlantic Forest regions. Other naturally infected species were detected, such as *Sa. chloropterus*, *Ae. Scapularis*, and *Ae. taeniorhinchus* [24,25,26].

Other signs of viral activity were recorded in the Amazon between 2017 and 2020. A new wave of viral expansion was detected in the Cerrado during the rainy season of 2020 in the state of GO [18,19,20,21,22,23,24,25,26,27]. The virus was confirmed in the Cerrado, northern MG, in August 2021. Dozens of epizootics in NHPs were recorded in the driest area of the state at the peak of the dry season, outside the usual seasonal transmission period in extra-Amazonian areas of Brazil, which lasts from December to May (the rainy season) [13,15,28].

The primary vectors of YFV in tropical rainforests (average annual rainfall from 1500 to 2000 mm), such as the Amazon and Atlantic Forests, have long been known to be mosquitoes of the genus *Haemagogus*, whilst other mosquitoes act as secondary vectors during outbreaks [24,29,30,31,32]. However, the Cerrado biome serves as a “corridor” through which YFV spreads from the Amazon to the Atlantic Forests. Unlike in these rainforests, the climatic conditions in the Cerrado hamper the filling of tree holes with rainwater—the breeding sites for *Haemagogus* mosquitoes during most of the year. Little is known about the primary vectors in semiarid regions (average annual rainfall of less than 800 mm), such as the Cerrado, or the means by which viral transmission and spatial spread are maintained between transmission seasons. Events that maintain virus viability during the dry season in this biome, when *Haemagogus* vectors essentially disappear, remain unknown [33]. In light of this, in the current study, we carried out an entomological-virological investigation in areas recently affected by YF in search of mosquitoes involved in the transmission of YFV in the semiarid region of MG. 

## 2. Materials and Methods

### 2.1. Study Area

**Figure 1:**
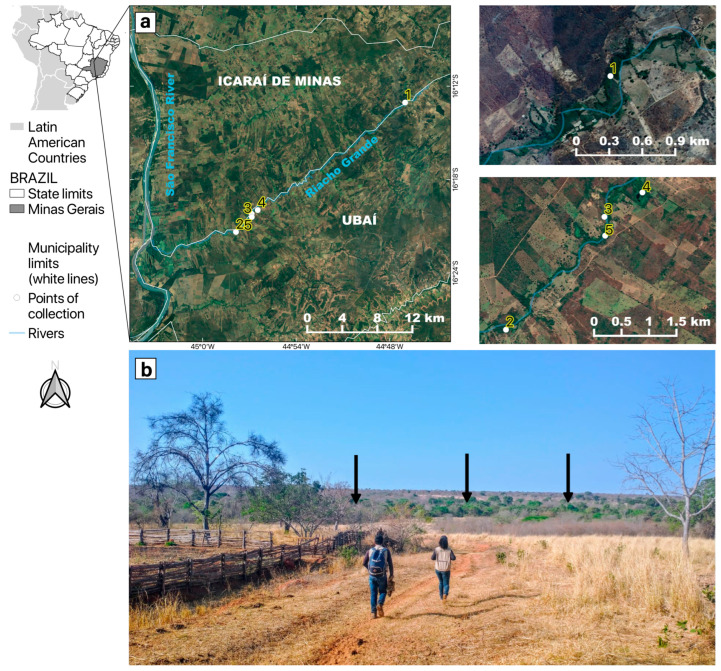
Location of the study area in the neighboring municipalities of Icaraí de Minas and Ubaí, in the northern part of Minas Gerais. (**a**) Satellite pictures showing mosquito sampling points (1 to 5) along the “Riacho Grande” river bordered by a narrow riparian forest surrounded by a highly fragmented landscape, composed of pastures and plantations. (**b**) The arid landscape where the study was conducted. The black arrows show that the only green area was the riparian forest where mosquitoes were sampled.

This study encompassed five mosquito sampling sites in riparian forest fragments of “Riacho Grande”, a small river of the São Francisco river watershed located in the rural zone that divides the two municipalities of Ubaí and Icaraí de Minas in northern MG, Brazil (Figure 1), where several YFV epizootics were reported between August and September 2021 [27]. The study area was inserted in an anthropized matrix, surrounded by pastures, with low tree coverage, and was essentially restricted to a narrow corridor of riparian forest. On average, the tree canopies in this area are 15 m high at most. The study area was situated in the ecotone between the Cerrado and Caatinga, another Brazilian endemic and dry biome, with a predominance of plant physiognomies and species typical of the Cerrado [34].

The regional climate is classified as tropical semiarid (Aw type) according to Köppen (1936) [35], with two well-defined seasons: a sharp dry season from March to October, and a rainy season from November to February. The months with the lowest rainfall are June to September, with an average historical rainfall of 3 to 18 mm (https://www.climatempo.com.br/climatologia/4080/icaraideminas-mg, accessed on 20 December 2022). The 2021 epizootics and the entomological sampling described here took place during the dry season.

### 2.2. Sampling, Taxonomic Classification, and RNA Extraction from Mosquitoes

Mosquito collections were carried out over 12 days in 2021, between 5 August and 1 October, around five sampling points where dead *Alouatta caraya* were found (Figure 1a). Adult mosquito captures were made during the day (07:30 a.m. to 5:30 p.m.) and around twilight (5:30 p.m. to 7:00 p.m.). Daytime collections were conducted using entomological nets and oral aspirators [24]; one light-baited Shannon trap including two collectors was used for the twilight sampling [36].

During the last six days of collection (26 September to 1 October), captures were simultaneously conducted on the ground and in the tree canopy. Temperature and relative humidity were recorded using thermohydrometers every 15 min and each time a mosquito was captured.

Adult mosquitoes were stocked in liquid nitrogen (−196 °C) and sent to the laboratory. Taxonomic identification was carried out on a cold table at −20 °C using a stereoscopic microscope and dichotomous keys [37,38,39,40]. Mosquitoes of the same species from the same collection point were grouped into pools of up to 10 individuals in 500 µL of L-15 with 20% of fetal bovine serum culture medium and homogenized using a Loccus L-Beader 24^®^ tissue homogenizer in bead tubes [24]. After centrifugation (9600× *g*, 5 min, at 4 °C), RNA was extracted from 140 µL of supernatant using the Qiagen RNA Viral Kit following the manufacturer’s recommendations. Only non-blood-fed mosquitoes were analyzed.

In order to increase sampling of tree-hole mosquitoes (e.g., *Haemagogus* sp.), twelve ovitraps, each with two wooden paddles, were hung from tree canopies at sampling point 5 (Figure 1), remaining exposed for 15 days, as previously described [41]. Point 5 was selected because the largest number of carcasses of *Alouatta caraya* were found there. The paddles were examined under a stereoscopic microscope searching for mosquito eggs. 

### 2.3. Virus Detection

YFV infection was screened within the RNA extracted from each pool of mosquitoes, in triplicate, with a RT-qPCR assay [42]. Viral RNA was reverse-transcribed and amplified using the GoTaq Probe 1-Step RT-qPCR System (Promega) in an Applied Biosystems Quantstudio-3 instrument. Primers, probes, and RT-qPCR conditions followed previously published protocols targeting the 5′-noncoding region of the YFV genome [42].

### 2.4. Genome Sequencing

The RNA of all YFV-positive samples was submitted to a cDNA synthesis protocol using LunaScript™ RT SuperMix Kit (NEB, Ipswich, MA, USA) following the manufacturer’s instructions. Next, a multiplex tiling PCR was performed using the previously published YFV primers [23] in a 40-cycle PCR amplification protocol (denaturation: 95 °C/15 s; annealing/extension: 65 °C/5 min) using Q5 high-fidelity DNA polymerase (NEB, Ipswich, MA, USA). PCR amplicons were purified with 1× AMPure XP beads (Beckman Coulter, Indianapolis, IN, USA) and cleaned-up PCR product concentrations were measured using a QuantiFluor^®^ dsDNA System assay kit on a Quantus™ Fluorometer (Promega, Madison, WI, USA). The DNA library preparation was performed using the Ligation Sequencing kit SQK-LSK309 (Oxford Nanopore Technologies, Oxford, United Kingdom) and the Native barcoding kit (EXP-NBD104 and EXP-NBD114; Oxford Nanopore Technologies, Oxford, UK). The sequencing library (2 positive samples and a negative control per run) was loaded onto an R9.4 flow cell (Oxford Nanopore Technologies, Oxford, UK) and sequenced between 6 and 18 h using MiNKNOW software (Oxford Nanopore Technologies, Oxford, UK). The RAMPART (Version 1.2.0, ARTIC Network, Oxford, UK) package was used to monitor coverage depth and genome completion. The resulting Fast5 files were base-called and demultiplexed using Guppy (Version 4.4.2, Oxford Nanopore Technologies, Oxford, United Kingdom). Variant calling and consensus genome assembly were carried out with Medaka (Version 1.0.3, Oxford Nanopore Technologies, Oxford, UK). The sequence used as a reference has the GenBank accession number JF912190.

### 2.5. Phylogenetic Analysis

To perform phylogenetic analysis, all near-complete YFV sequences (*n* = 929, sequences > 8 kb excluding sequences from vaccine and patents) were selected from GenBank (available on https://www.ncbi.nlm.nih.gov/, accessed on 20 December 2022). Metadata such as sample collection dates and geographic coordinates were retrieved from GenBank files or gathered manually from genome-associated publications. The genomes recovered here (*n* = 2) combined with 929 genomes from NCBI were aligned with MAFFT v.7.480 [43]. A maximum-likelihood tree was inferred using the FastTree 2.1.5 plugin [44] implemented in Geneious R10. This program uses the neighbor-joining method to get an approximate initial tree, and then minimal evolution methods to reduce the length of the tree, before maximum likelihood to further improve the tree. Next, the branch support was estimated by a Shimodaira–Hasegawa-like test [45]. The new genome sequences were sent to the NCBI GenBank database under accession numbers OQ572695 and OQ572696.

## 3. Results

In total, 917 adult female mosquitoes from 13 taxa were collected (Table 1 and Table 2) and divided into 165 pools. Notably, mosquitoes of *Sabethes* genus accounted for 95% (*n* = 674) of the 709 specimens collected during daytime (Table 1). The relative abundance of *Sa. chloropterus* (53%, 376 individuals) was the highest, followed by *Sa. albiprivus* (42%, 298 individuals) (Table 1). In the twilight period, 208 specimens were collected, and *Culex* sp. was the most abundant taxon (Table 2).

No eggs were found in the ovitraps. Interestingly, mosquitoes of the genus *Haemagogus*, the primary vector of YF in Brazil, were not found, either in adult or in immature stages.

**Figure 2:**
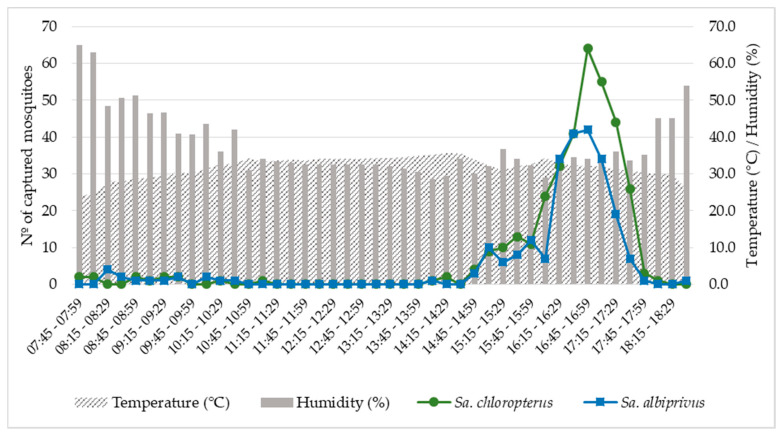
Biting activity of *Sabethes chloropterus* and *Sabethes albiprivus*, calculated by the sum of individuals captured in 15-min intervals, from 26 September to 1 October, 2021 in Ubaí and Icaraí de Minas, northern MG. Average temperatures and relative humidities recorded during the sampling period are also shown.

*Sa. chloropterus* and *Sa. albiprivus* exhibited a strong peak of biting activity at the end of the day, from 4:00 p.m. to 5:45 p.m., with a greater number of adults captured from 4:45 p.m. to 4:59 p.m., coinciding with trends of slightly increasing relative humidity (from 30 to 35%) and declining temperature (from 34 °C to 30 °C). Few individuals were captured during the morning (*n* = 28) or during twilight (*n* = 6) (Figure 2; Supplementary Material S1).

**Figure 3:**
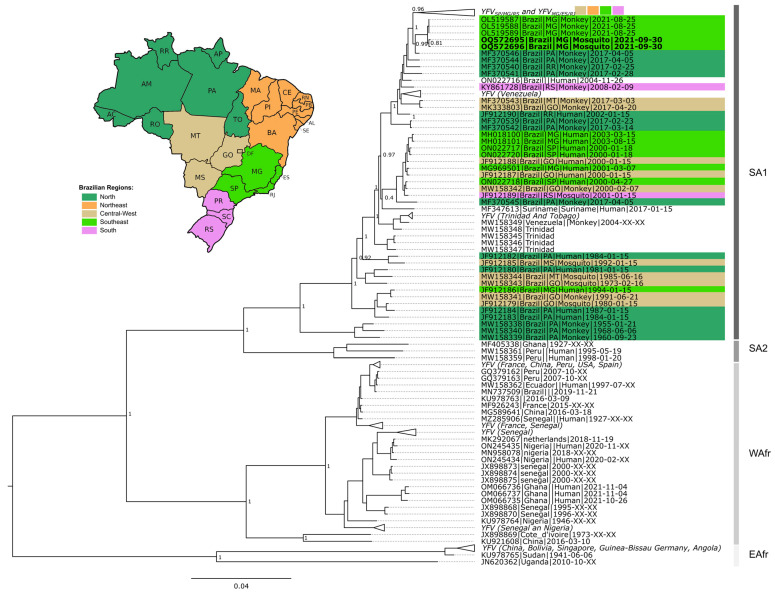
Phylogenetic tree of YFV based on 931 near-complete genomes. The green collapsed group at the top of the tree includes YFV_MG/ES/RJ_ and YFV_MG/SP_ clades. The host type (human, NHP and mosquitoes) are indicated after tip name by a corresponding symbol. South America 1, South America 2, West Africa, and East Africa genotypes are indicated. Brazilian genomes are highlighted according to geographic region. Extra-Amazonian waves are indicated by braces. Genomes generated in this study are in bold (OQ572695 and OQ572696). Support values as well as all sequences and metadata used are presented in Supplementary Material S2.

Among the 165 tested pools, three were positive for YFV: two of *Sa. chloropterus* (MIR = 5.3) and one of *Sa. albiprivus* (MIR = 3.3) (Table 1 and Table 3). It was possible to sequence a near-complete genome of YFV detected in the two pools of *Sa. chloropterus* (code X247 and X313, Table 3). Phylogenetic analysis revealed that the viral genome could be assigned to the South American 1 genotype, sub-clade 1E, which has been circulating in Brazil in recent years. Furthermore, they clustered along a viral sub-lineage that circulated during 2017 in Pará state, Amazon region, and which was previously named YFV_PA/MG_ [27] (Figure 3; Supplementary Materials S2 and S3). Due to the high Ct value found in the only infected *Sa. albiprivus* pool (code X214, Table 3), efforts to sequence fragments of that genome failed.

## 4. Discussion

In this study, we obtained data from an entomological investigation following NHP deaths due to YF in one of the most arid regions of MG, at the peak of the dry season in the Cerrado biome. These were conditions—hitherto scarcely studied—that were apparently unsuitable for YFV circulation and maintenance in nature. Interestingly, we described the natural infection of *Sa. chloropterus* in the Cerrado of MG for the first time. We also produced the first report on YFV in *Sa. albiprivus* in Brazil. These were found to be the dominant species, whose level of biting activity in late afternoons was unprecedented. These results may help to fill an important gap in the understanding of YFV dispersion mechanisms in the extra-Amazonian region, especially when YFV crosses the Cerrado area, and also when it spreads outside of the usual transmission period—the rainy season from December to May [13].

Natural YFV infections in *Sa. chloropterus* were reported several times in tropical rain forests of Central America (Guatemala and Panama) in the 1950s [46,47]. In addition, there are records of natural infections in riparian forests of the Cerrado in the Central-West Region of Brazil in 1992 [5], and in the Atlantic Forest in 2019 [24], both during the rainy season. In this paper, in addition to describing the first natural infection in *Sa. chloropterus* from the semiarid Cerrado of Southeast Brazil, in MG, we suggest that this species may be a primary vector of YFV in the region because it seemed capable of initiating and maintaining the epizootics, even during the dry season. The concept of vector capacity was developed to describe the capacity of a population of vectors to transmit a pathogen in a given space and time [48,49]. Four aspects of this concept relating to *Sa. chloropterus,* incorporating our findings and certain biological characteristics of the species, allow us to develop a hypothesis explaining the main role of *Sa. chloropterus* in the maintenance of YFV outside the seasonal period in the semiarid Cerrado. (1) Its large relative abundance (>50%) compared with samplings taken in other regions, where it did not exceed 3% of the captured specimens [24,25,50,51,52,53,54]. The vector abundance is directly related to the number of bites per host per day, and to the number of females per host, both of which are biological parameters of the vector capacity equation. (2) Higher MIR, together with the high number of copies of YFV RNA found in positive pools (mean CT between 19.0 and 21.0), near to mean CTs found in YFV-infected pools of *Haemagogus* mosquitoes (ranging between 17.0 and 20.0) in recent outbreaks in Southeast region during the rainy season [12,24]. A high number of copies of viral RNA indirectly indicates viral dissemination and transmission in mosquito bodies and enhances vector competence [55,56], another biological parameter of the vector capacity equation. (3) Its high longevity [57,58]—another component of the vectorial capacity equation—increases the chances of a female becoming infected, amplifying the virus and transmitting it to different hosts. (4) The complete absence of *Haemagogus* mosquitoes, even in ovitraps, which are considered highly sensitive to detect their presence [43,59,60,61,62]. These observations reinforce the probable main role of *Sa. chloropterus* in the maintenance of YFV in this scenario. The region’s inherent semiarid climate, accentuated by the prevailing drought during the epizootics and mosquito sampling time, apparently limits the growth of some tree-hole mosquitoes, such as *Haemagogus janthinomys* and *Hg. leucocelaenus*. However, *Sa. chloropterus* has previously been described as resistant to drought in tropical forests, in the sense that its population density can maintain a certain stability even under unfavorable circumstances for the vast majority of mosquitoes due to the particularities of its breeding sites. *Sa. chloropterus* lays eggs in very specific tree holes with a large internal cavity and very small lateral opening, which reduce evaporation and retain water much longer than the hollows with wide openings typically used by *Haemagogus* mosquitoes [5,63,64].

Despite having been proved competent to transmit YFV [65], *Sa. albiprivus* has previously been found naturally infected only once, in Argentina [66]. The present study presents the second report of natural YFV infection in this species and the first in Brazil. *Sa. albiprivus* has been frequently collected in Brazil in different biomes, including small fragments of anthropized forests, such as the riparian forests in the Cerrado of the Central-West region, and also the Atlantic Forest areas of RJ and MG, where *Sa. albiprivus* has shown high relative abundance and was the most abundant *Sabethes* species during recent YF outbreaks [24,67]. The oviposition behavior of *Sa. albiprivus* was recently recorded, and includes females throwing eggs into Sapucaia (*Lecythis pisonis*) nuts, simulating a tree hole with a small opening, which is its natural larval habitat [68]. As in *Sa. chloropterus,* this behavior results in resistance to drought, which could allow such mosquitoes to contribute to the maintenance of YFV in adverse conditions for *Haemagogus*.

Surprisingly, the biting activity of both captured *Sabethes* species abruptly peaked during the same time interval (between 4:30 and 5:30 p.m.). Thus, it seems that they both respond to similar conditions, such as temperature, humidity, and luminosity at the end of the afternoon. Outside this peak period, no biting activity was observed throughout the day, except on very rare occasions. To the best of our knowledge, this is the first time that this hematophagy pattern has been described for these *Sabethes* species. In the tropical rainforests of Panama, the biting activity of *Sa. chloropterus* was found to be practically constant from 9:00 a.m. to 3:00 p.m. (rainy season sampling) with a slight peak between 2:00 to 3:00 p.m. [64]. Similar figures were obtained in Trinidad in the rainy season by the authors of [69], who also identified a bimodal peak pattern in the dry season, firstly from 08:00 a.m. to 10:00 a.m., and secondly from 12:00 p.m. to 2:00 p.m. [69]. We hypothesize that the biting peak of *Sa. chloropterus* recorded at the end of the afternoon in the semiarid Cerrado could be related to extreme climatic conditions in this region. It seems that the biting behavior of both *Sa. chloropterus* and *Sa. albiprivus* responds to a trend of decreasing temperature after the daily maximum is reached, and to slightly increasing relative humidity after the minimum daily value is attained. It may be that the reduction in solar incidence and its position almost on the horizon line also influence biting activity (although we did not measure this variable) in combination with the trends of decreasing temperature and increasing humidity. Differences in mosquito biting peaks during the day are probably related to climatic variations in wind, temperature, humidity, and sunlight, in addition to endogenous stimuli [70,71]. It is recommended that this peak of activity be considered during future entomological investigations in the region, even at the risk of undersampling these species. It is equally important to conduct mosquito collections as soon as possible after the detection of epizootics.

Our data suggest that there is no accented vertical stratification of biting activity for either of the recorded *Sabethes* species. Vertical stratification is most evident in tropical rainforests where the trees are taller and the forest is denser, and there are differences in climatic conditions between the two strata (canopy vs. ground) [64,72]. In less dense forests and in places with prolonged drought, such as the riparian forests of the semiarid Cerrado, these differences tend to diminish or disappear [67]. This profile of biting activity in mosquitoes traditionally considered primatophilic and acrodendrophilic (e.g., *Haemagogus* and *Sabethes*) increases the chances of transmission of YFV to humans entering or approaching forested areas.

The phylogenetic analysis of the generated YFV genomes reinforces the hypothesis that the investigated epizootic is due to a new wave of viral expansion from the Amazon region [27], i.e., different from the one that caused the 2014–2021 outbreak, named YFV_PA/MG_ [15] (A bayesian phylogenetic tree is presented in Supplementary Material S3 [73,74,75,76,77]). Opportunities for sequencing near-complete YFV genomes detected in mosquitoes, especially *Sa. chloropterus*, are rare, and offer the chance to make important contributions to the understanding of viral dynamics in different hosts.

In summary, although YFV transmission is more common in the rainy season (seasonal period), the circulation of the virus can also occur at the height of the dry season in previously poorly-studied biomes such as the Cerrado, which serves as a corridor during waves of viral expansion. The low diversity of species and, most importantly, the absence of *Haemagogus* sp. during the samplings suggest that other species are able to sustain YFV circulation in semiarid regions. In this sense, the demonstration that *Sa. chloropterus* and *Sa. albiprivus* remain abundant, even in the dry season in areas dominated by the semiarid Cerrado, and that they potentially may be involved in YFV transmission until climatic conditions, favor other well-known vector species, which highlights the complexity of the dynamics of viral circulation and the spread in such circumstances. Moreover, the surveillance of vectors and epizootics and the intensification of vaccination in risk areas should be priorities to avoid human cases, even in time periods and environmental conditions considered unsuitable for viral circulation.

## Figures and Tables

**Table 1:** Relative abundance and total numbers of Culicidae species collected in the daytime period (7:30 a.m. to 5:00 p.m.) using entomological nets and oral aspirators, in Ubaí and Icaraí de Minas, northern MG state.

Species	Nº of Mosquitoes					
	Ground (%)	Canopy (%)	Total	Rel. Abund.%	Pools Tested (Positive)	MIR *
*Sabethes chloropterus* (von Humboldt, 1819)	194 (51.6)	182 (48.4)	376	53.0	74 (2)	5.3
*Sabethes albiprivus* Theobald, 1903	208 (69.8)	90 (30.2)	298	42.0	61 (1)	3.3
*Aedes scapularis* (Rondani, 1848)	13 (100)	0 (0)	13	1.8	2 (0)	0
*Wyeomyia* sp.	7 (100)	0 (0)	7	0.9	1 (0)	0
*Aedes albopictus* (Skuse, 1894)	4 (80)	1 (20)	5	0.7	1 (0)	0
*Psorophora ferox* (von Humboldt, 1819)	4 (100)	0 (0)	4	0.5	1 (0)	0
*Wyeomyia melanocephala* Dyar & Knab, 1906	3 (100)	0 (0)	3	0.4	1 (0)	0
*Coquillettidia venezuelensis* (Theobald, 1912)	3 (100)	0 (0)	3	0.4	1 (0)	0
Total	436 (61.5)	273 (38.5)	709	100	142 (3)	

* Minimum Infection Rate (MIR) =  nº of positive pools/nº of same species adults analyzed ×  1000; Rel. abund.: Relative abundance.

**Table 2:** Relative abundance and total numbers of Culicidae collected around twilight (5:30 p.m. to 7:00 p.m.) with Shannon trap, in Ubaí and Icaraí de Minas, northern MG state.

Species	Nº of Mosquitoes	Rel. Abund.%	Pools Tested (Positive)
*Culex (Melanoconion)* sp.	83	39.9	4 (0)
*Culex* sp.	76	36.5	8 (0)
*Coquillettidia venezuelensis* (Theobald, 1912)	10	4.8	1 (0)
*Culex* (*Culex*) sp.	8	3.8	1 (0)
*Uranotaenia geometrica* Theobald, 1901	8	3.8	1 (0)
*Anopheles evansae* (Brèthes, 1926)	4	1.9	1 (0)
*Mansonia titillans* (Walker, 1848)	4	1.9	1 (0)
*Sabethes chloropterus* (von Humboldt, 1819)	4	1.9	1 (0)
*Anopheles* (*Nyssorhynchus*) sp.	4	1.9	1 (0)
*Aedes albopictus* [Skuse, 1894]	3	1.4	1 (0)
*Uranotaenia nataliae* Lynch Arribálzaga, 1981	2	0.9	1 (0)
*Sabethes albiprivus* Theobald, 1903	1	0.4	1 (0)
*Aedes scapularis* [Rondani, 1848]	1	0.4	1 (0)
Total	208	100	23 (0)

Legend. Rel. abund.: Relative abundance.

**Table 3:** Description of YFV-positive mosquito pools.

Pool Code	Collection Date	Municipality	Location	Latitude, Longitude	Species	nº of Mosquitoes		CT Mean
						Ground	Canopy	
X214	26 September 2021	Ubaí	Lagoa Seca	−16.340069, −44.947525	*Sa. albiprivus*	5	0	28
X247	30 September 2021	Icaraí de Minas	Vila Nova	−16.356050, −44.965317 −16.355514, −44.963906	*Sa. chloropterus*	5	0	19
X313	30 September 2021	Icaraí de Minas	Vila Nova		*Sa. chloropterus*	0	5	21

Legend: CT = Cycle Threshold.

## Data Availability

Not applicable.

## Supplementary Materials

The following supporting information can be downloaded at: https://www.mdpi.com/article/10.3390/v15030757/s1, Material S1: Table S1—Number of *Sabethes* mosquitoes captured every 15 minutes, from September 26 to 1 October 2021, in Ubaí and Icaraí de Minas, northern MG. The average temperature and relative humidity measured during the sampling are shown.; Material S2: Figure S1—Phylogenetic tree of YFV based on 962 near-complete genomes. Genomes generated in this study are highlighted in Red (GenBank accession numbers OQ572695 and OQ572696). Support values are indicated. Table S2—List of all sequences used as well as metadata, including geographic information; Material S3: Bayesian phylogenetic analyses. Figure S2—Time-scaled phylogenetic tree of 100 complete and near-complete South American genotype I genome sequences and the two new genomes generated in our study sampled in Minas Gerais. Colors represent different sampling locations according to the legend on the left of the tree. Genomes reported in the presented study are colored in red.
