# Supplementary material for: Yellow Fever Virus Maintained by Sabethes Mosquitoes during the Dry Season in Cerrado, a Semiarid Region of Brazil, in 2021
Source: Viruses. 2023 Mar 15;15(3):757. doi: 10.3390/v15030757 (PMC10058068; doi:10.3390/v15030757)
Supplement: Supplementary file 1 [file viruses-15-00757-s001.zip › Suppl. material S1 2147632.pdf]

Supplementary Table S1: Number of *Sabethes* mosquitoes captured every 15 minutes, from September 26 to October 1, 2021, in Ubaí and Icaraí de Minas, northern MG. The average temperature and relative humidity measured during the sampling are shown.

Time	*Sa. chloropterus*	*Sa. albiprivus*	Mean humidity (%)	Mean temperature (°C)
07:45 - 07:59	2	0	65,0	24,1
08:00 - 08:14	2	0	63,0	24,4
08:15 - 08:29	0	4	48,3	28,2
08:30 - 08:44	0	2	50,5	27,8
08:45 - 08:59	2	1	51,3	28,8
09:00 - 09:14	1	1	46,5	29,0
09:15 - 09:29	2	1	46,7	29,5
09:30 - 09:44	2	2	40,8	30,6
09:45 - 09:59	0	0	40,7	30,0
10:00 - 10:14	0	2	43,5	31,5
10:15 - 10:29	1	1	36,0	32,6
10:30 - 10:44	0	1	42,0	32,8
10:45 - 10:59	0	0	31,0	34,2
11:00 - 11:14	1	0	34,0	33,2
11:15 - 11:29	0	0	33,5	33,6
11:30 - 11:44	0	0	33,0	33,9
11:45 - 11:59	0	0	32,5	33,7
12:00 - 12:14	0	0	32,5	34,0
12:15 - 12:29	0	0	32,5	34,0
12:30 - 12:44	0	0	32,5	34,0
12:45 - 12:59	0	0	32,5	34,0
13:00 - 13:14	0	0	32,5	34,2
13:15 - 13:29	0	0	32,0	34,3
13:30 - 13:44	0	0	31,5	34,5
13:45 - 13:59	0	0	30,5	34,9
14:00 - 14:14	1	1	28,5	35,2
14:15 - 14:29	2	0	29,5	35,7
14:30 - 14:44	0	0	34,0	35,7
14:45 - 14:59	4	3	30,0	33,8
15:00 - 15:14	9	10	32,1	32,4
15:15 - 15:29	10	6	36,8	31,0
15:30 - 15:44	13	8	34,0	32,0
15:45 - 15:59	11	12	32,3	32,5
16:00 - 16:14	24	7	28,9	34,2
16:15 - 16:29	32	34	33,5	33,0
16:30 - 16:44	41	41	34,4	32,4
16:45 - 16:59	64	42	34,0	32,1
17:00 - 17:14	55	34	33,8	32,1
17:15 - 17:29	44	19	36,0	31,3
17:30 - 17:44	26	7	33,7	30,9
17:45 - 17:59	3	1	35,2	30,3

18:00 - 18:14	1	0	45,0	29,9
18:15 - 18:29	0	0	45,0	29,9
18:30 - 18:45	0	1	54,0	26,0
