# Supplementary material for: Yellow Fever Virus Maintained by Sabethes Mosquitoes during the Dry Season in Cerrado, a Semiarid Region of Brazil, in 2021
Source: Viruses. 2023 Mar 15;15(3):757. doi: 10.3390/v15030757 (PMC10058068; doi:10.3390/v15030757)
Supplement: Supplementary file 1 [file viruses-15-00757-s001.zip › Suppl. material S2 2147632pdf.pdf]

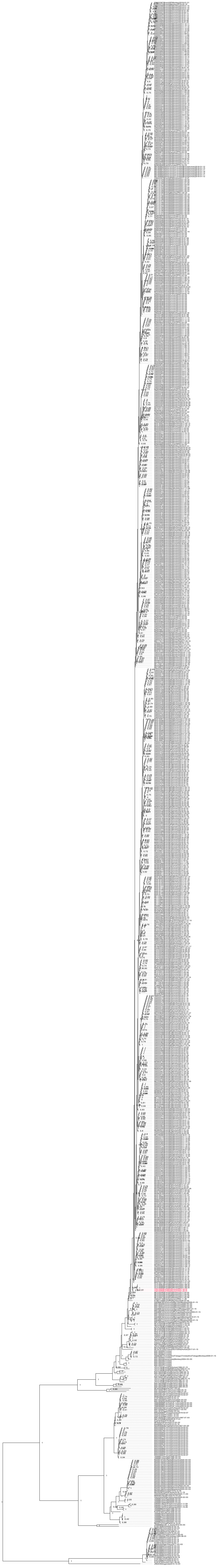

Suppl. Figure S1: Phylogenetic tree of YFV based on 962 near-complete genomes. Genomes generated in this study are highlighted in Red (GenBank acession numbers: OQ572695 and OQ572696). Support values are indicated.

Suppl. Table S2: List of all sequences used as well as metadata, including geographic information.

Name	Collection_date	Host	isolate	Country	State	Friendly Name
HM582851	2009-01-15	Monkey		TrinidadAndTobago	TrinidadAndTobago	HM582851|TrinidadAndTobago|Monkey|2009-01-15
JF912179	1980-01-15	Mosquito		Brazil	GO	JF912179|Brazil|GO|Mosquito|1980-01-15
JF912180	1981-01-15	Human		Brazil	PA	JF912180|Brazil|PA|Human|1981-01-15
JF912182	1984-01-15	Human		Brazil	PA	JF912182|Brazil|PA|Human|1984-01-15
JF912183	1984-01-15	Human		Brazil	PA	JF912183|Brazil|PA|Human|1984-01-15
JF912184	1987-01-15	Human		Brazil	PA	JF912184|Brazil|PA|Human|1987-01-15
JF912185	1992-01-15	Mosquito		Brazil	MS	JF912185|Brazil|MS|Mosquito|1992-01-15
JF912186	1994-01-15	Human		Brazil	MG	JF912186|Brazil|MG|Human|1994-01-15
JF912187	2000-01-15	Human		Brazil	GO	JF912187|Brazil|GO|Human|2000-01-15
JF912188	2000-01-15	Human		Brazil	GO	JF912188|Brazil|GO|Human|2000-01-15
JF912189	2001-01-15	Mosquito		Brazil	RS	JF912189|Brazil|RS|Mosquito|2001-01-15
JF912190	2002-01-15	Human		Brazil	RR	JF912190|Brazil|RR|Human|2002-01-15
KM388814	2005-01-15	Human		Venezuela	Portuguesa	KM388814|Venezuela|Portuguesa|Human|2005-01-15
KM388815	2007-01-15	Monkey		Venezuela	Apure	KM388815|Venezuela|Apure|Monkey|2007-01-15
KM388816	2010-01-15	Monkey		Venezuela	Monagas	KM388816|Venezuela|Monagas|Monkey|2010-01-15
KM388817	2004-01-15	Monkey		Venezuela	Guarico	KM388817|Venezuela|Guarico|Monkey|2004-01-15
KM388818	2006-01-15	Monkey		Venezuela	Barinas	KM388818|Venezuela|Barinas|Monkey|2006-01-15
KY861728	2008-02-09	Monkey		Brazil	RS	KY861728|Brazil|RS|Monkey|2008-02-09
KY885000	2017-02-20	Monkey		Brazil	ES	KY885000|Brazil|ES|Monkey|2017-02-20
KY885001	2017-02-22	Monkey		Brazil	ES	KY885001|Brazil|ES|Monkey|2017-02-22
MF170968	2017-04-13	Human		Brazil	ES	MF170968|Brazil|ES|Human|2017-04-13
MF170969	2017-03-18	Human		Brazil	ES	MF170969|Brazil|ES|Human|2017-03-18
MF170970	2017-03-21	Human		Brazil	ES	MF170970|Brazil|ES|Human|2017-03-21
MF170971	2017-01-30	Monkey		Brazil	MG	MF170971|Brazil|MG|Monkey|2017-01-30
MF170972	2017-03-31	Human		Brazil	ES	MF170972|Brazil|ES|Human|2017-03-31
MF170973	2017-04-10	Human		Brazil	ES	MF170973|Brazil|ES|Human|2017-04-10
MF170974	2017-03-29	Human		Brazil	ES	MF170974|Brazil|ES|Human|2017-03-29
MF170975	2017-03-03	Human		Brazil	ES	MF170975|Brazil|ES|Human|2017-03-03
MF170976	2017-01-25	Human		Brazil	ES	MF170976|Brazil|ES|Human|2017-01-25
MF170977	2017-02-10	Human		Brazil	ES	MF170977|Brazil|ES|Human|2017-02-10
MF170978	2017-03-28	Human		Brazil	ES	MF170978|Brazil|ES|Human|2017-03-28
MF170979	2017-04-06	Human		Brazil	ES	MF170979|Brazil|ES|Human|2017-04-06
MF170980	2017-03-23	Human		Brazil	ES	MF170980|Brazil|ES|Human|2017-03-23

MF170981	2017-04-05	Human	Brazil	ES	MF170981|Brazil|ES|Human|2017-04-05
MF347613	2017-01-15	Human	Suriname	Suriname	MF347613|Suriname|Suriname|Human|2017-01-15
MF370530	2017-01-15	Mosquito	Brazil	MG	MF370530|Brazil|MG|Mosquito|2017-01-15
MF370531	2017-02-28	Human	Brazil	ES	MF370531|Brazil|ES|Human|2017-02-28
MF370532	2017-01-10	Human	Brazil	MG	MF370532|Brazil|MG|Human|2017-01-10
MF370533	2017-01-11	Human	Brazil	MG	MF370533|Brazil|MG|Human|2017-01-11
MF370534	2016-04-05	Monkey	Brazil	MG	MF370534|Brazil|MG|Monkey|2016-04-05
MF370535	2016-08-30	Monkey	Brazil	MG	MF370535|Brazil|MG|Monkey|2016-08-30
MF370536	2016-09-02	Monkey	Brazil	MG	MF370536|Brazil|MG|Monkey|2016-09-02
MF370537	2017-01-17	Monkey	Brazil	ES	MF370537|Brazil|ES|Monkey|2017-01-17
MF370538	2017-01-20	Monkey	Brazil	MG	MF370538|Brazil|MG|Monkey|2017-01-20
MF370539	2017-02-23	Monkey	Brazil	PA	MF370539|Brazil|PA|Monkey|2017-02-23
MF370540	2017-02-25	Monkey	Brazil	RR	MF370540|Brazil|RR|Monkey|2017-02-25
MF370541	2017-02-28	Monkey	Brazil	PA	MF370541|Brazil|PA|Monkey|2017-02-28
MF370542	2017-03-14	Monkey	Brazil	PA	MF370542|Brazil|PA|Monkey|2017-03-14
MF370543	2017-03-03	Monkey	Brazil	MT	MF370543|Brazil|MT|Monkey|2017-03-03
MF370544	2017-04-05	Monkey	Brazil	PA	MF370544|Brazil|PA|Monkey|2017-04-05
MF370545	2017-04-05	Monkey	Brazil	PA	MF370545|Brazil|PA|Monkey|2017-04-05
MF370546	2017-04-05	Monkey	Brazil	PA	MF370546|Brazil|PA|Monkey|2017-04-05
MF370547	2017-03-27	Monkey	Brazil	RJ	MF370547|Brazil|RJ|Monkey|2017-03-27
MF370548	2017-04-20	Monkey	Brazil	RJ	MF370548|Brazil|RJ|Monkey|2017-04-20
MF370549	2015-08-26	Monkey	Brazil	MG	MF370549|Brazil|MG|Monkey|2015-08-26
MF423373	2017-02-21	Mosquito	Brazil	ES	MF423373|Brazil|ES|Mosquito|2017-02-21
MF423374	2017-02-23	Mosquito	Brazil	ES	MF423374|Brazil|ES|Mosquito|2017-02-23
MF423375	2017-04-04	Monkey	Brazil	RJ	MF423375|Brazil|RJ|Monkey|2017-04-04
MF423376	2017-04-13	Monkey	Brazil	RJ	MF423376|Brazil|RJ|Monkey|2017-04-13
MF423377	2017-04-19	Monkey	Brazil	RJ	MF423377|Brazil|RJ|Monkey|2017-04-19
MF423378	2017-04-19	Monkey	Brazil	RJ	MF423378|Brazil|RJ|Monkey|2017-04-19
MF434851	2017-04-25	Human	Brazil	RJ	MF434851|Brazil|RJ|Human|2017-04-25
MF465805	2017-01-17	Human	Brazil	Brazil	MF465805|Brazil|Brazil|Human|2017-01-17
MF538782	2017-03-16	Human	Brazil	RJ	MF538782|Brazil|RJ|Human|2017-03-16
MF538783	2017-03-18	Human	Brazil	RJ	MF538783|Brazil|RJ|Human|2017-03-18
MF538784	2017-02-26	Human	Brazil	RJ	MF538784|Brazil|RJ|Human|2017-02-26
MF538785	2017-04-21	Monkey	Brazil	RJ	MF538785|Brazil|RJ|Monkey|2017-04-21

MF538786	2017-06-05	Monkey	Brazil	RJ	MF538786|Brazil|RJ|Monkey|2017-06-05
MG550109	2017-04-18	Human	Brazil	RJ	MG550109|Brazil|RJ|Human|2017-04-18
MG969501	2001-03-07	Human	Brazil	MG	MG969501|Brazil|MG|Human|2001-03-07
MH018064	2017-02-15	Monkey	Brazil	MG	MH018064|Brazil|MG|Monkey|2017-02-15
MH018065	2017-02-14	Monkey	Brazil	MG	MH018065|Brazil|MG|Monkey|2017-02-14
MH018066	2017-02-15	Monkey	Brazil	MG	MH018066|Brazil|MG|Monkey|2017-02-15
MH018067	2017-02-13	Monkey	Brazil	MG	MH018067|Brazil|MG|Monkey|2017-02-13
MH018068	2017-01-09	Human	Brazil	MG	MH018068|Brazil|MG|Human|2017-01-09
MH018069	2017-01-14	Human	Brazil	MG	MH018069|Brazil|MG|Human|2017-01-14
MH018070	2017-01-21	Human	Brazil	MG	MH018070|Brazil|MG|Human|2017-01-21
MH018071	2017-01-25	Human	Brazil	MG	MH018071|Brazil|MG|Human|2017-01-25
MH018072	2017-01-07	Human	Brazil	MG	MH018072|Brazil|MG|Human|2017-01-07
MH018073	2017-01-20	Human	Brazil	MG	MH018073|Brazil|MG|Human|2017-01-20
MH018074	2017-01-14	Human	Brazil	MG	MH018074|Brazil|MG|Human|2017-01-14
MH018075	2017-01-18	Human	Brazil	MG	MH018075|Brazil|MG|Human|2017-01-18
MH018076	2017-01-19	Human	Brazil	MG	MH018076|Brazil|MG|Human|2017-01-19
MH018077	2017-01-07	Human	Brazil	MG	MH018077|Brazil|MG|Human|2017-01-07
MH018078	2017-01-18	Human	Brazil	MG	MH018078|Brazil|MG|Human|2017-01-18
MH018079	2017-01-28	Human	Brazil	MG	MH018079|Brazil|MG|Human|2017-01-28
MH018080	2017-01-28	Human	Brazil	MG	MH018080|Brazil|MG|Human|2017-01-28
MH018081	2017-02-22	Monkey	Brazil	MG	MH018081|Brazil|MG|Monkey|2017-02-22
MH018082	2017-02-20	Monkey	Brazil	MG	MH018082|Brazil|MG|Monkey|2017-02-20
MH018083	2017-02-17	Monkey	Brazil	MG	MH018083|Brazil|MG|Monkey|2017-02-17
MH018084	2017-02-09	Monkey	Brazil	MG	MH018084|Brazil|MG|Monkey|2017-02-09
MH018085	2017-01-13	Human	Brazil	MG	MH018085|Brazil|MG|Human|2017-01-13
MH018086	2017-01-19	Human	Brazil	MG	MH018086|Brazil|MG|Human|2017-01-19
MH018087	2017-01-10	Human	Brazil	MG	MH018087|Brazil|MG|Human|2017-01-10
MH018088	2017-01-21	Human	Brazil	MG	MH018088|Brazil|MG|Human|2017-01-21
MH018089	2017-01-02	Human	Brazil	MG	MH018089|Brazil|MG|Human|2017-01-02
MH018090	2017-01-12	Human	Brazil	MG	MH018090|Brazil|MG|Human|2017-01-12
MH018091	2017-01-30	Human	Brazil	MG	MH018091|Brazil|MG|Human|2017-01-30
MH018092	2017-01-27	Human	Brazil	MG	MH018092|Brazil|MG|Human|2017-01-27
MH018093	2017-01-20	Human	Brazil	MG	MH018093|Brazil|MG|Human|2017-01-20
MH018094	2017-01-30	Human	Brazil	MG	MH018094|Brazil|MG|Human|2017-01-30

MH018095	2017-01-19	Monkey	Brazil	MG	MH018095|Brazil|MG|Monkey|2017-01-19
MH018096	2017-01-13	Monkey	Brazil	MG	MH018096|Brazil|MG|Monkey|2017-01-13
MH018097	2017-01-25	Monkey	Brazil	MG	MH018097|Brazil|MG|Monkey|2017-01-25
MH018098	2017-01-25	Monkey	Brazil	MG	MH018098|Brazil|MG|Monkey|2017-01-25
MH018099	2017-03-10	Monkey	Brazil	BA	MH018099|Brazil|BA|Monkey|2017-03-10
MH018100	2003-03-15	Human	Brazil	MG	MH018100|Brazil|MG|Human|2003-03-15
MH018101	2003-08-15	Human	Brazil	MG	MH018101|Brazil|MG|Human|2003-08-15
MH018102	2017-01-31	Monkey	Brazil	ES	MH018102|Brazil|ES|Monkey|2017-01-31
MH018103	2017-01-30	Human	Brazil	MG	MH018103|Brazil|MG|Human|2017-01-30
MH018104	2017-01-30	Human	Brazil	MG	MH018104|Brazil|MG|Human|2017-01-30
MH018105	2017-01-28	Human	Brazil	MG	MH018105|Brazil|MG|Human|2017-01-28
MH018106	2017-01-13	Monkey	Brazil	MG	MH018106|Brazil|MG|Monkey|2017-01-13
MH018107	2017-02-22	Human	Brazil	ES	MH018107|Brazil|ES|Human|2017-02-22
MH018108	2017-03-10	Human	Brazil	ES	MH018108|Brazil|ES|Human|2017-03-10
MH018109	2017-03-08	Monkey	Brazil	ES	MH018109|Brazil|ES|Monkey|2017-03-08
MH018110	2017-03-09	Monkey	Brazil	ES	MH018110|Brazil|ES|Monkey|2017-03-09
MH018111	2017-04-10	Human	Brazil	ES	MH018111|Brazil|ES|Human|2017-04-10
MH018112	2017-04-17	Monkey	Brazil	RJ	MH018112|Brazil|RJ|Monkey|2017-04-17
MH018113	2017-04-22	Human	Brazil	RJ	MH018113|Brazil|RJ|Human|2017-04-22
MH018114	2017-01-24	Monkey	Brazil	ES	MH018114|Brazil|ES|Monkey|2017-01-24
MH018115	2017-01-24	Monkey	Brazil	ES	MH018115|Brazil|ES|Monkey|2017-01-24
MH030049	2017-08-17	Monkey	Brazil	SP	MH030049|Brazil|SP|Monkey|2017-08-17
MH030050	2017-08-16	Monkey	Brazil	SP	MH030050|Brazil|SP|Monkey|2017-08-16
MH030051	2017-09-15	Monkey	Brazil	SP	MH030051|Brazil|SP|Monkey|2017-09-15
MH030052	2017-09-15	Monkey	Brazil	SP	MH030052|Brazil|SP|Monkey|2017-09-15
MH030053	2017-08-30	Monkey	Brazil	SP	MH030053|Brazil|SP|Monkey|2017-08-30
MH030054	2017-09-15	Monkey	Brazil	SP	MH030054|Brazil|SP|Monkey|2017-09-15
MH030055	2017-09-11	Monkey	Brazil	SP	MH030055|Brazil|SP|Monkey|2017-09-11
MH030056	2017-09-12	Monkey	Brazil	SP	MH030056|Brazil|SP|Monkey|2017-09-12
MH030057	2017-09-27	Monkey	Brazil	SP	MH030057|Brazil|SP|Monkey|2017-09-27
MH030058	2017-09-26	Monkey	Brazil	SP	MH030058|Brazil|SP|Monkey|2017-09-26
MH030059	2017-09-21	Monkey	Brazil	SP	MH030059|Brazil|SP|Monkey|2017-09-21
MH030060	2017-09-27	Monkey	Brazil	SP	MH030060|Brazil|SP|Monkey|2017-09-27
MH030061	2017-09-21	Monkey	Brazil	SP	MH030061|Brazil|SP|Monkey|2017-09-21

MH030062	2017-09-19	Monkey	Brazil	SP	MH030062|Brazil|SP|Monkey|2017-09-19
MH030063	2017-10-15	Monkey	Brazil	SP	MH030063|Brazil|SP|Monkey|2017-10-15
MH030064	2017-10-15	Monkey	Brazil	SP	MH030064|Brazil|SP|Monkey|2017-10-15
MH030065	2017-10-15	Monkey	Brazil	SP	MH030065|Brazil|SP|Monkey|2017-10-15
MH030066	2017-10-15	Monkey	Brazil	SP	MH030066|Brazil|SP|Monkey|2017-10-15
MH030067	2017-10-15	Monkey	Brazil	SP	MH030067|Brazil|SP|Monkey|2017-10-15
MH030068	2017-10-15	Monkey	Brazil	SP	MH030068|Brazil|SP|Monkey|2017-10-15
MH030069	2017-10-15	Monkey	Brazil	SP	MH030069|Brazil|SP|Monkey|2017-10-15
MH030070	2017-10-19	Monkey	Brazil	SP	MH030070|Brazil|SP|Monkey|2017-10-19
MH030071	2017-10-18	Monkey	Brazil	SP	MH030071|Brazil|SP|Monkey|2017-10-18
MH030072	2017-10-15	Monkey	Brazil	SP	MH030072|Brazil|SP|Monkey|2017-10-15
MH030073	2017-10-18	Monkey	Brazil	SP	MH030073|Brazil|SP|Monkey|2017-10-18
MH030074	2017-10-18	Monkey	Brazil	SP	MH030074|Brazil|SP|Monkey|2017-10-18
MH030075	2017-10-15	Monkey	Brazil	SP	MH030075|Brazil|SP|Monkey|2017-10-15
MH030076	2017-10-15	Monkey	Brazil	SP	MH030076|Brazil|SP|Monkey|2017-10-15
MH030077	2017-10-15	Monkey	Brazil	SP	MH030077|Brazil|SP|Monkey|2017-10-15
MH030078	2017-10-15	Monkey	Brazil	SP	MH030078|Brazil|SP|Monkey|2017-10-15
MH030079	2017-11-15	Monkey	Brazil	SP	MH030079|Brazil|SP|Monkey|2017-11-15
MH030080	2017-11-15	Monkey	Brazil	SP	MH030080|Brazil|SP|Monkey|2017-11-15
MH030081	2017-11-15	Monkey	Brazil	SP	MH030081|Brazil|SP|Monkey|2017-11-15
MH030082	2017-11-15	Monkey	Brazil	SP	MH030082|Brazil|SP|Monkey|2017-11-15
MH030083	2017-11-15	Monkey	Brazil	SP	MH030083|Brazil|SP|Monkey|2017-11-15
MH030084	2017-11-15	Monkey	Brazil	SP	MH030084|Brazil|SP|Monkey|2017-11-15
MH030085	2017-11-15	Monkey	Brazil	SP	MH030085|Brazil|SP|Monkey|2017-11-15
MH030086	2017-11-15	Monkey	Brazil	SP	MH030086|Brazil|SP|Monkey|2017-11-15
MH030087	2017-04-26	Human	Brazil	SP	MH030087|Brazil|SP|Human|2017-04-26
MH030088	2017-03-03	Human	Brazil	SP	MH030088|Brazil|SP|Human|2017-03-03
MH030089	2017-03-03	Human	Brazil	SP	MH030089|Brazil|SP|Human|2017-03-03
MH030090	2017-03-02	Human	Brazil	SP	MH030090|Brazil|SP|Human|2017-03-02
MH030091	2017-03-29	Human	Brazil	SP	MH030091|Brazil|SP|Human|2017-03-29
MH193173	2018-01-09	Monkey	Brazil	SP	MH193173|Brazil|SP|Monkey|2018-01-09
MH193174	2018-01-09	Human	Brazil	SP	MH193174|Brazil|SP|Human|2018-01-09
MH193175	2018-02-01	Monkey	Brazil	SP	MH193175|Brazil|SP|Monkey|2018-02-01
MH329655	2018-01-29	Mosquito	Brazil	MG	MH329655|Brazil|MG|Mosquito|2018-01-29

MH378284	2017-01-15	Human	Brazil	Brazil	MH378284|Brazil|Brazil|Human|2017-01-15
MH484423	2017-01-24	Monkey	Brazil	MG	MH484423|Brazil|MG|Monkey|2017-01-24
MH484424	2017-01-24	Human	Brazil	MG	MH484424|Brazil|MG|Human|2017-01-24
MH484425	2017-01-27	Human	Brazil	MG	MH484425|Brazil|MG|Human|2017-01-27
MH484426	2017-01-13	Monkey	Brazil	MG	MH484426|Brazil|MG|Monkey|2017-01-13
MH484427	2017-01-24	Monkey	Brazil	MG	MH484427|Brazil|MG|Monkey|2017-01-24
MH484428	2017-02-05	Human	Brazil	MG	MH484428|Brazil|MG|Human|2017-02-05
MH484429	2017-02-03	Monkey	Brazil	MG	MH484429|Brazil|MG|Monkey|2017-02-03
MH484430	2017-02-09	Monkey	Brazil	MG	MH484430|Brazil|MG|Monkey|2017-02-09
MH484431	2017-02-14	Monkey	Brazil	MG	MH484431|Brazil|MG|Monkey|2017-02-14
MH484432	2017-01-23	Monkey	Brazil	MG	MH484432|Brazil|MG|Monkey|2017-01-23
MH484433	2017-02-03	Monkey	Brazil	MG	MH484433|Brazil|MG|Monkey|2017-02-03
MH484434	2017-01-22	Human	Brazil	MG	MH484434|Brazil|MG|Human|2017-01-22
MH560359	2017-01-15	Human	Brazil	MG	MH560359|Brazil|MG|Human|2017-01-15
MH666056	2016-11-16	Monkey	Brazil	SP	MH666056|Brazil|SP|Monkey|2016-11-16
MH666057	2017-01-23	Monkey	Brazil	SP	MH666057|Brazil|SP|Monkey|2017-01-23
MH666058	2016-12-19	Monkey	Brazil	SP	MH666058|Brazil|SP|Monkey|2016-12-19
MH666059	2017-01-15	Mosquito	Brazil	SP	MH666059|Brazil|SP|Mosquito|2017-01-15
MH666060	2017-01-15	Mosquito	Brazil	SP	MH666060|Brazil|SP|Mosquito|2017-01-15
MK089775	2017-03-06	Mosquito	Brazil	RJ	MK089775|Brazil|RJ|Mosquito|2017-03-06
MK249065	2017-02-15	Human	Brazil	MG	MK249065|Brazil|MG|Human|2017-02-15
MK249066	2018-01-15	Monkey	Brazil	MG	MK249066|Brazil|MG|Monkey|2018-01-15
MK333798	2018-01-08	Human	Brazil	SP	MK333798|Brazil|SP|Human|2018-01-08
MK333799	2018-01-08	Human	Brazil	SP	MK333799|Brazil|SP|Human|2018-01-08
MK333800	2017-02-20	Monkey	Brazil	ES	MK333800|Brazil|ES|Monkey|2017-02-20
MK333801	2017-04-05	Mosquito	Brazil	ES	MK333801|Brazil|ES|Mosquito|2017-04-05
MK333802	2017-04-05	Mosquito	Brazil	ES	MK333802|Brazil|ES|Mosquito|2017-04-05
MK333803	2017-04-20	Monkey	Brazil	GO	MK333803|Brazil|GO|Monkey|2017-04-20
MK333804	2015-08-17	Monkey	Brazil	GO	MK333804|Brazil|GO|Monkey|2015-08-17
MK333805	2018-02-07	Mosquito	Brazil	RJ	MK333805|Brazil|RJ|Mosquito|2018-02-07
MK333806	2018-01-27	Mosquito	Brazil	MG	MK333806|Brazil|MG|Mosquito|2018-01-27
MK333807	2018-01-18	Mosquito	Brazil	MG	MK333807|Brazil|MG|Mosquito|2018-01-18
MK333808	2018-01-29	Mosquito	Brazil	MG	MK333808|Brazil|MG|Mosquito|2018-01-29
MK333809	2018-01-19	Mosquito	Brazil	RJ	MK333809|Brazil|RJ|Mosquito|2018-01-19

MK533792	2019-01-10	Monkey	Brazil	RJ	MK533792|Brazil|RJ|Monkey|2019-01-10
MK728873	2017-03-04	Human	Brazil	GO	MK728873|Brazil|GO|Human|2017-03-04
MK760660	2018-01-11	Human	Netherlands	TravelledBrazil	MK760660|Netherlands|TraveBrazil|Human|2018-01-11
MK760661	2018-01-11	Human	Netherlands	TravelledBrazil	MK760661|Netherlands|TraveBrazil|Human|2018-01-11
MK760662	2018-01-12	Human	Netherlands	TravelledBrazil	MK760662|Netherlands|TraveBrazil|Human|2018-01-12
MK760663	2018-01-13	Human	Netherlands	TravelledBrazil	MK760663|Netherlands|TraveBrazil|Human|2018-01-13
MK760664	2018-01-14	Human	Netherlands	TravelledBrazil	MK760664|Netherlands|TraveBrazil|Human|2018-01-14
MK760665	2018-01-19	Human	Netherlands	TravelledBrazil	MK760665|Netherlands|TraveBrazil|Human|2018-01-19
MK760666	2018-01-12	Human	Netherlands	TravelledBrazil	MK760666|Netherlands|TraveBrazil|Human|2018-01-12
MK882599	2017-02-09	Human	Brazil	ES	MK882599|Brazil|ES|Human|2017-02-09
MK882600	2018-04-24	Human	Brazil	RJ	MK882600|Brazil|RJ|Human|2018-04-24
MK882601	2017-01-24	Human	Brazil	ES	MK882601|Brazil|ES|Human|2017-01-24
MK882602	2017-01-31	Human	Brazil	ES	MK882602|Brazil|ES|Human|2017-01-31
MK882603	2018-04-24	Human	Brazil	RJ	MK882603|Brazil|RJ|Human|2018-04-24
MK882604	2017-02-16	Human	Brazil	ES	MK882604|Brazil|ES|Human|2017-02-16
MK882605	2017-03-09	Human	Brazil	RJ	MK882605|Brazil|RJ|Human|2017-03-09
MK882606	2017-03-07	Human	Brazil	RJ	MK882606|Brazil|RJ|Human|2017-03-07
MK882607	2017-03-09	Human	Brazil	RJ	MK882607|Brazil|RJ|Human|2017-03-09
MK882608	2017-03-12	Human	Brazil	RJ	MK882608|Brazil|RJ|Human|2017-03-12
MK882609	2017-03-13	Human	Brazil	ES	MK882609|Brazil|ES|Human|2017-03-13
MK882610	2017-03-06	Human	Brazil	ES	MK882610|Brazil|ES|Human|2017-03-06
MK882611	2017-03-08	Human	Brazil	ES	MK882611|Brazil|ES|Human|2017-03-08
MK882612	2017-03-20	Human	Brazil	ES	MK882612|Brazil|ES|Human|2017-03-20
MK882613	2017-03-27	Human	Brazil	RJ	MK882613|Brazil|RJ|Human|2017-03-27
MK882614	2017-04-17	Human	Brazil	RJ	MK882614|Brazil|RJ|Human|2017-04-17
MK882615	2017-03-27	Human	Brazil	RJ	MK882615|Brazil|RJ|Human|2017-03-27
MK882616	2017-04-17	Human	Brazil	RJ	MK882616|Brazil|RJ|Human|2017-04-17
MK882617	2017-11-28	Human	Brazil	RJ	MK882617|Brazil|RJ|Human|2017-11-28
MK882618	2018-01-22	Human	Brazil	RJ	MK882618|Brazil|RJ|Human|2018-01-22
MK882619	2018-01-25	Human	Brazil	RJ	MK882619|Brazil|RJ|Human|2018-01-25
MK882621	2018-02-05	Human	Brazil	RJ	MK882621|Brazil|RJ|Human|2018-02-05
MN117917	2017-02-15	Mosquito	Brazil	SP	MN117917|Brazil|SP|Mosquito|2017-02-15
MN604280	2017-03-10	Monkey	Brazil	BA	MN604280|Brazil|BA|Monkey|2017-03-10
MN604281	2017-03-13	Monkey	Brazil	BA	MN604281|Brazil|BA|Monkey|2017-03-13

MN604282	2017-03-16	Monkey	Brazil	BA	MN604282|Brazil|BA|Monkey|2017-03-16
MN604283	2017-03-15	Monkey	Brazil	BA	MN604283|Brazil|BA|Monkey|2017-03-15
MN604284	2017-03-15	Human	Brazil	BA	MN604284|Brazil|BA|Human|2017-03-15
MN604285	2017-04-05	Monkey	Brazil	BA	MN604285|Brazil|BA|Monkey|2017-04-05
MN604286	2017-04-07	Monkey	Brazil	BA	MN604286|Brazil|BA|Monkey|2017-04-07
MN604287	2017-03-17	Monkey	Brazil	BA	MN604287|Brazil|BA|Monkey|2017-03-17
MN643077	2018-01-16	Human	Brazil	RJ	MN643077|Brazil|RJ|Human|2018-01-16
MN643078	2018-01-25	Human	Brazil	RJ	MN643078|Brazil|RJ|Human|2018-01-25
MN643079	2018-01-26	Human	Brazil	RJ	MN643079|Brazil|RJ|Human|2018-01-26
MN643080	2018-02-17	Human	Brazil	RJ	MN643080|Brazil|RJ|Human|2018-02-17
MN643081	2018-02-19	Human	Brazil	RJ	MN643081|Brazil|RJ|Human|2018-02-19
MN643082	2018-02-16	Human	Brazil	RJ	MN643082|Brazil|RJ|Human|2018-02-16
MN643083	2018-02-25	Human	Brazil	RJ	MN643083|Brazil|RJ|Human|2018-02-25
MN643084	2018-02-25	Human	Brazil	RJ	MN643084|Brazil|RJ|Human|2018-02-25
MN643085	2018-02-11	Human	Brazil	RJ	MN643085|Brazil|RJ|Human|2018-02-11
MN643086	2018-02-14	Human	Brazil	RJ	MN643086|Brazil|RJ|Human|2018-02-14
MN643087	2018-02-13	Human	Brazil	RJ	MN643087|Brazil|RJ|Human|2018-02-13
MN643088	2017-04-19	Monkey	Brazil	RJ	MN643088|Brazil|RJ|Monkey|2017-04-19
MN643089	2017-10-19	Monkey	Brazil	RJ	MN643089|Brazil|RJ|Monkey|2017-10-19
MN643090	2018-02-04	Monkey	Brazil	RJ	MN643090|Brazil|RJ|Monkey|2018-02-04
MN643091	2018-02-07	Monkey	Brazil	RJ	MN643091|Brazil|RJ|Monkey|2018-02-07
MN643092	2018-02-07	Monkey	Brazil	RJ	MN643092|Brazil|RJ|Monkey|2018-02-07
MN737509	2019-11-21		Brazil		MN737509|Brazil|||2019-11-21
MT497520	2016-10-10	Monkey	Brazil	SP	MT497520|Brazil|SP|Monkey|2016-10-10
MT497521	2016-10-13	Monkey	Brazil	SP	MT497521|Brazil|SP|Monkey|2016-10-13
MT497522	2016-11-15	Monkey	Brazil	SP	MT497522|Brazil|SP|Monkey|2016-11-15
MT497523	2016-11-16	Monkey	Brazil	SP	MT497523|Brazil|SP|Monkey|2016-11-16
MT497524	2016-12-12	Monkey	Brazil	SP	MT497524|Brazil|SP|Monkey|2016-12-12
MT497525	2017-01-21	Monkey	Brazil	SP	MT497525|Brazil|SP|Monkey|2017-01-21
MW034590	2016-11-01	Monkey	Brazil	SP	MW034590|Brazil|SP|Monkey|2016-11-01
MW158338	1955-01-21	Monkey	Brazil		MW158338|Brazil||Monkey|1955-01-21
MW158339	1960-09-23	Monkey	Brazil		MW158339|Brazil||Monkey|1960-09-23
MW158340	1968-06-06	Monkey	Brazil		MW158340|Brazil||Monkey|1968-06-06
MW158341	1991-06-21	Monkey	Brazil		MW158341|Brazil||Monkey|1991-06-21

MW158342	2000-02-07	Monkey		Brazil	MW158342|Brazil||Monkey|2000-02-07
MW158343	1973-02-16	Mosquito		Brazil	MW158343|Brazil||Mosquito|1973-02-16
MW158344	1985-06-16	Mosquito		Brazil	MW158344|Brazil||Mosquito|1985-06-16
MW158345	1988-12-14	Mosquito		Trinidad and Tobago	MW158345|Trinidad and Tobago||Mosquito|1988-12-14
MW158346	1989-01-20	Mosquito		Trinidad and Tobago	MW158346|Trinidad and Tobago||Mosquito|1989-01-20
MW158347	1989-02-22	Monkey		Trinidad and Tobago	MW158347|Trinidad and Tobago||Monkey|1989-02-22
MW158348	1995-XX-XX	Mosquito		Trinidad and Tobago	MW158348|Trinidad and Tobago||Mosquito|1995-XX-XX
MW158349	2004-XX-XX	Monkey		Venezuela	MW158349|Venezuela||Monkey|2004-XX-XX
MW158350	2004-XX-XX	Monkey		Venezuela	MW158350|Venezuela||Monkey|2004-XX-XX
MW158351	2005-XX-XX	Monkey		Venezuela	MW158351|Venezuela||Monkey|2005-XX-XX
MW158352	2005-XX-XX	Monkey		Venezuela	MW158352|Venezuela||Monkey|2005-XX-XX
MW158353	2005-XX-XX	Human		Venezuela	MW158353|Venezuela||Human|2005-XX-XX
MW158354	2005-XX-XX	Human		Venezuela	MW158354|Venezuela||Human|2005-XX-XX
MW158355	2006-XX-XX	Monkey		Venezuela	MW158355|Venezuela||Monkey|2006-XX-XX
MW158356	2007-XX-XX	Monkey		Venezuela	MW158356|Venezuela||Monkey|2007-XX-XX
MW158357	2010-XX-XX	Monkey		Venezuela	MW158357|Venezuela||Monkey|2010-XX-XX
MW158358	2004-XX-XX	Human		Peru	MW158358|Peru||Human|2004-XX-XX
MW158359	1998-01-20	Human		Peru	MW158359|Peru||Human|1998-01-20
MW158360	2011-03-XX	Human		Peru	MW158360|Peru||Human|2011-03-XX
MW158361	1995-05-19	Human		Peru	MW158361|Peru||Human|1995-05-19
MW158362	1997-07-XX	Human		Ecuador	MW158362|Ecuador||Human|1997-07-XX
MW158363	1966-XX-XX	Human		USA	MW158363|USA||Human|1966-XX-XX
MW158364	2009-01-22	Mosquito		Trinidad and Tobago	MW158364|Trinidad and Tobago||Mosquito|2009-01-22
MW158365	2008-12-05	Mosquito		Trinidad and Tobago	MW158365|Trinidad and Tobago||Mosquito|2008-12-05
MW158366	2009-01-16	Mosquito		Trinidad and Tobago	MW158366|Trinidad and Tobago||Mosquito|2009-01-16
MW158367	2009-01-16	Mosquito		Trinidad and Tobago	MW158367|Trinidad and Tobago||Mosquito|2009-01-16
MW249074	2019-02-07	Human	7D_P3	France	MW249074|France||Human|2019-02-07
MW249075	2019-02-07	Human	17D_P4	France	MW249075|France||Human|2019-02-07
MW249076	2019-02-07	Human	17D_P5	France	MW249076|France||Human|2019-02-07
MW249077	2019-02-07	Human	FNV_P3	France	MW249077|France||Human|2019-02-07
MW249078	2019-02-07	Human	FNV_P4	France	MW249078|France||Human|2019-02-07
MW249079	2019-02-07	Human	FNV_P5	France	MW249079|France||Human|2019-02-07
MW249080	2019-02-07	Human	Stamaril_2002	France	MW249080|France||Human|2019-02-07
MW249081	2019-02-07	Human	FNV-NS1	France	MW249081|France||Human|2019-02-07

MW249082	2019-02-07	Human	FNV-N2	France		MW249082|France||Human|2019-02-07
MW249083	2019-02-07	Human	FNV-N3	France		MW249083|France||Human|2019-02-07
MW249084	2019-02-07	Human	FNV-NS4	France		MW249084|France||Human|2019-02-07
MW249085	2019-02-07	Human	FNV-S5	France		MW249085|France||Human|2019-02-07
MW249086	2019-02-07	Human	FNV-N6	France		MW249086|France||Human|2019-02-07
MW249087	2019-02-07	Human	FNV-S7	France		MW249087|France||Human|2019-02-07
MW249088	2019-02-07	Human	FNV-S8	France		MW249088|France||Human|2019-02-07
MW249089	2019-02-07	Human	FNV-NS9	France		MW249089|France||Human|2019-02-07
MW249090	2019-02-07	Human	FNV-NS10	France		MW249090|France||Human|2019-02-07
MW249091	2019-02-07	Human	FNV-S11	France		MW249091|France||Human|2019-02-07
MW249092	2019-02-07	Human	FNV-NS13	France		MW249092|France||Human|2019-02-07
MW249093	2019-02-07	Human	FNV-N14	France		MW249093|France||Human|2019-02-07
MW249094	2019-02-07	Human	FNV-NS15	France		MW249094|France||Human|2019-02-07
MW249095	2019-02-07	Human	FNV-N12	France		MW249095|France||Human|2019-02-07
MW308134	2018-10-26	Human	YFV084FIG	Brazil	SP	MW308134|Brazil|SP|Human|2018-10-26
MW308135	2019-01-20	Human	YFV085FIG	Brazil	SP	MW308135|Brazil|SP|Human|2019-01-20
MZ285906	1927-XX-XX	Human		Senegal		MZ285906|Senegal||Human|1927-XX-XX
MZ595182	2020-XX-XX	Mosquito	PM322117	Senegal		MZ595182|Senegal||Mosquito|2020-XX-XX
MZ595183	2020-XX-XX	Mosquito	PM322188	Senegal		MZ595183|Senegal||Mosquito|2020-XX-XX
MZ595184	2020-XX-XX	Mosquito	PM352481	Senegal		MZ595184|Senegal||Mosquito|2020-XX-XX
MZ595185	2020-XX-XX	Mosquito	PM352628	Senegal		MZ595185|Senegal||Mosquito|2020-XX-XX
MZ595186	2020-XX-XX	Mosquito	PM352640	Senegal		MZ595186|Senegal||Mosquito|2020-XX-XX
MZ595187	2020-XX-XX	Mosquito	PM352649	Senegal		MZ595187|Senegal||Mosquito|2020-XX-XX
MZ595188	2020-XX-XX	Mosquito	PM352652	Senegal		MZ595188|Senegal||Mosquito|2020-XX-XX
MZ595189	2020-XX-XX	Mosquito	PM352662	Senegal		MZ595189|Senegal||Mosquito|2020-XX-XX
MZ595190	2020-XX-XX	Mosquito	PM352721	Senegal		MZ595190|Senegal||Mosquito|2020-XX-XX
MZ595191	2020-XX-XX	Mosquito	PM352818	Senegal		MZ595191|Senegal||Mosquito|2020-XX-XX
MZ595192	2020-XX-XX	Mosquito	PM352820	Senegal		MZ595192|Senegal||Mosquito|2020-XX-XX
MZ595193	2020-XX-XX	Mosquito	PM352859	Senegal		MZ595193|Senegal||Mosquito|2020-XX-XX
MZ595194	2020-XX-XX	Mosquito	PM352903	Senegal		MZ595194|Senegal||Mosquito|2020-XX-XX
MZ595195	2020-XX-XX	Mosquito	PM352943	Senegal		MZ595195|Senegal||Mosquito|2020-XX-XX
MZ595196	2020-XX-XX	Mosquito	PM352951	Senegal		MZ595196|Senegal||Mosquito|2020-XX-XX
MZ595197	2020-XX-XX	Mosquito	PM352972	Senegal		MZ595197|Senegal||Mosquito|2020-XX-XX
MZ595198	2020-XX-XX	Mosquito	PM353136	Senegal		MZ595198|Senegal||Mosquito|2020-XX-XX

MZ595199	2020-XX-XX	Mosquito	PM353171	Senegal	MZ595199|Senegal||Mosquito|2020-XX-XX
MZ595203	2020-XX-XX	Human	SH329086	Senegal	MZ595203|Senegal||Human|2020-XX-XX
MZ604838	2019-XX-XX	Human		Brazil	MZ604838|Brazil||Human|2019-XX-XX
MZ604839	2019-XX-XX	Human		Brazil	MZ604839|Brazil||Human|2019-XX-XX
MZ604840	2018-XX-XX	Human		Brazil	MZ604840|Brazil||Human|2018-XX-XX
MZ604841	2019-XX-XX	Human		Brazil	MZ604841|Brazil||Human|2019-XX-XX
MZ604842	2018-XX-XX	Human		Brazil	MZ604842|Brazil||Human|2018-XX-XX
MZ604843	2019-XX-XX	Human		Brazil	MZ604843|Brazil||Human|2019-XX-XX
MZ604844	2019-XX-XX	Human		Brazil	MZ604844|Brazil||Human|2019-XX-XX
MZ604845	2018-XX-XX	Human		Brazil	MZ604845|Brazil||Human|2018-XX-XX
MZ604846	2019-XX-XX	Human		Brazil	MZ604846|Brazil||Human|2019-XX-XX
MZ604847	2019-XX-XX	Human		Brazil	MZ604847|Brazil||Human|2019-XX-XX
MZ604848	2019-XX-XX	Human		Brazil	MZ604848|Brazil||Human|2019-XX-XX
MZ604849	2019-XX-XX	Human		Brazil	MZ604849|Brazil||Human|2019-XX-XX
MZ604850	2019-XX-XX	Human		Brazil	MZ604850|Brazil||Human|2019-XX-XX
MZ604851	2019-XX-XX	Human		Brazil	MZ604851|Brazil||Human|2019-XX-XX
MZ604852	2019-XX-XX	Human		Brazil	MZ604852|Brazil||Human|2019-XX-XX
MZ604853	2019-XX-XX	Human		Brazil	MZ604853|Brazil||Human|2019-XX-XX
MZ604854	2019-XX-XX	Human		Brazil	MZ604854|Brazil||Human|2019-XX-XX
MZ604855	2019-XX-XX	Human		Brazil	MZ604855|Brazil||Human|2019-XX-XX
MZ604856	2018-XX-XX	Human		Brazil	MZ604856|Brazil||Human|2018-XX-XX
MZ604857	2019-XX-XX	Human		Brazil	MZ604857|Brazil||Human|2019-XX-XX
MZ604858	2019-XX-XX	Human		Brazil	MZ604858|Brazil||Human|2019-XX-XX
MZ604859	2018-XX-XX	Human		Brazil	MZ604859|Brazil||Human|2018-XX-XX
MZ604860	2019-XX-XX	Human		Brazil	MZ604860|Brazil||Human|2019-XX-XX
MZ604861	2018-XX-XX	Human		Brazil	MZ604861|Brazil||Human|2018-XX-XX
MZ604862	2018-XX-XX	Human		Brazil	MZ604862|Brazil||Human|2018-XX-XX
MZ604863	2019-XX-XX	Human		Brazil	MZ604863|Brazil||Human|2019-XX-XX
MZ604864	2019-XX-XX	Human		Brazil	MZ604864|Brazil||Human|2019-XX-XX
MZ604865	2018-XX-XX	Human		Brazil	MZ604865|Brazil||Human|2018-XX-XX
MZ604866	2018-XX-XX	Human		Brazil	MZ604866|Brazil||Human|2018-XX-XX
MZ604867	2019-XX-XX	Human		Brazil	MZ604867|Brazil||Human|2019-XX-XX
MZ604868	2018-XX-XX	Human		Brazil	MZ604868|Brazil||Human|2018-XX-XX
MZ604869	2019-XX-XX	Human		Brazil	MZ604869|Brazil||Human|2019-XX-XX

MZ604870	2018-XX-XX	Human		Brazil		MZ604870|Brazil||Human|2018-XX-XX
MZ604871	2019-XX-XX	Human		Brazil		MZ604871|Brazil||Human|2019-XX-XX
MZ604872	2018-XX-XX	Human		Brazil		MZ604872|Brazil||Human|2018-XX-XX
MZ604873	2018-XX-XX	Human		Brazil		MZ604873|Brazil||Human|2018-XX-XX
MZ604874	2019-XX-XX	Human		Brazil		MZ604874|Brazil||Human|2019-XX-XX
MZ604875	2019-XX-XX	Human		Brazil		MZ604875|Brazil||Human|2019-XX-XX
MZ604876	2018-XX-XX	Human		Brazil		MZ604876|Brazil||Human|2018-XX-XX
MZ712127	2021-03-04	Monkey	Andre_da_Rocha_02	Brazil	RS	MZ712127|Brazil|RS|Monkey|2021-03-04
MZ712128	2021-02-22	Monkey	Barracao_02	Brazil	RS	MZ712128|Brazil|RS|Monkey|2021-02-22
MZ712129	2021-02-22	Monkey	Barracao_03	Brazil	RS	MZ712129|Brazil|RS|Monkey|2021-02-22
MZ712130	2021-02-22	Monkey	Barracao_04	Brazil		MZ712130|Brazil||Monkey|2021-02-22
MZ712131	2021-02-19	Monkey	Esmeralda_01	Brazil	RS	MZ712131|Brazil|RS|Monkey|2021-02-19
MZ712132	2021-02-24	Monkey	Esmeralda_02	Brazil	RS	MZ712132|Brazil|RS|Monkey|2021-02-24
MZ712133	2021-02-25	Monkey	Esmeralda_03	Brazil	RS	MZ712133|Brazil|RS|Monkey|2021-02-25
MZ712134	2021-02-25	Monkey	Esmeralda_04	Brazil	RS	MZ712134|Brazil|RS|Monkey|2021-02-25
MZ712135	2021-03-08	Monkey	Ipe_01	Brazil	RS	MZ712135|Brazil|RS|Monkey|2021-03-08
MZ712136	2021-02-08	Monkey	Monte_Alegre_dos_Campos_01	Brazil	RS	MZ712136|Brazil|RS|Monkey|2021-02-08
MZ712137	2021-02-26	Monkey	Monte_Alegre_dos_Campos_02	Brazil	RS	MZ712137|Brazil|RS|Monkey|2021-02-26
MZ712138	2021-03-03	Monkey	Monte_Alegre_dos_Campos_03	Brazil	RS	MZ712138|Brazil|RS|Monkey|2021-03-03
MZ712139	2021-02-20	Monkey	Muitos_Capoes_01	Brazil	RS	MZ712139|Brazil|RS|Monkey|2021-02-20
MZ712140	2021-01-25	Monkey	Pinhal_da_Serra_01	Brazil	RS	MZ712140|Brazil|RS|Monkey|2021-01-25
MZ712141	2021-02-03	Monkey	Pinhal_da_Serra_02	Brazil	RS	MZ712141|Brazil|RS|Monkey|2021-02-03
MZ712142	2021-02-03	Monkey	Pinhal_da_Serra_03	Brazil	RS	MZ712142|Brazil|RS|Monkey|2021-02-03
MZ712143	2021-02-08	Monkey	Pinhal_da_Serra_05	Brazil	RS	MZ712143|Brazil|RS|Monkey|2021-02-08
MZ712144	2021-02-11	Monkey	Pinhal_da_Serra_07	Brazil	RS	MZ712144|Brazil|RS|Monkey|2021-02-11
MZ712145	2021-02-19	Monkey	Pinhal_da_Serra_08	Brazil	RS	MZ712145|Brazil|RS|Monkey|2021-02-19
MZ712146	2021-02-19	Monkey	Pinhal_da_Serra_09	Brazil	RS	MZ712146|Brazil|RS|Monkey|2021-02-19
MZ712147	2021-02-22	Monkey	Vacaria_01	Brazil	RS	MZ712147|Brazil|RS|Monkey|2021-02-22
MZ712148	2021-02-22	Monkey	Vacaria_02	Brazil	RS	MZ712148|Brazil|RS|Monkey|2021-02-22
MZ712149	2021-02-24	Monkey	Vacaria_04	Brazil	RS	MZ712149|Brazil|RS|Monkey|2021-02-24
OL519587	2021-08-25	Monkey	MG66-L	Brazil	MG	OL519587|Brazil|MG|Monkey|2021-08-25
OL519588	2021-08-25	Monkey	MG66-S	Brazil	MG	OL519588|Brazil|MG|Monkey|2021-08-25
OL519589	2021-08-25	Monkey	MG66-k	Brazil	MG	OL519589|Brazil|MG|Monkey|2021-08-25
OM066735	2021-10-26	Human		Ghana		OM066735|Ghana||Human|2021-10-26

OM066736	2021-11-04	Human		Ghana		OM066736|Ghana||Human|2021-11-04
OM066737	2021-11-04	Human		Ghana		OM066737|Ghana||Human|2021-11-04
OM938988	2016-XX-XX	Cell_culture	MSLFDV02508	France		OM938988|France||Cell_culture|2016-XX-XX
OM938989	2018-XX-XX	Cell_culture	DSFDV02794	France		OM938989|France||Cell_culture|2018-XX-XX
OM938990	2019-XX-XX	Cell_culture	DSFDV02926	France		OM938990|France||Cell_culture|2019-XX-XX
ON022238	2017-01-24	Monkey	IAL-13	Brazil	ES	ON022238|Brazil|ES|Monkey|2017-01-24
ON022239	2018-01-17	Human	0158/18 FA	Brazil	MG	ON022239|Brazil|MG|Human|2018-01-17
ON022240	2018-01-05	Monkey	0042/18 IV	Brazil	MG	ON022240|Brazil|MG|Monkey|2018-01-05
ON022241	2018-01-02	Monkey	0053/18 IV	Brazil	MG	ON022241|Brazil|MG|Monkey|2018-01-02
ON022242	2018-01-15	Human	0100/18 FA	Brazil	MG	ON022242|Brazil|MG|Human|2018-01-15
ON022243	2018-01-08	Monkey	0051/18 IV	Brazil	MG	ON022243|Brazil|MG|Monkey|2018-01-08
ON022244	2018-01-17	Human	0154/18 FA	Brazil	MG	ON022244|Brazil|MG|Human|2018-01-17
ON022245	2018-01-05	Human	0031/18 FA	Brazil	MG	ON022245|Brazil|MG|Human|2018-01-05
ON022246	2018-01-09	Human	0044/18 FA	Brazil	MG	ON022246|Brazil|MG|Human|2018-01-09
ON022247	2018-01-13	Human	0071/18 FA	Brazil	MG	ON022247|Brazil|MG|Human|2018-01-13
ON022248	2018-01-15	Human	0107/18 FA	Brazil	MG	ON022248|Brazil|MG|Human|2018-01-15
ON022249	2018-01-16	Human	0129/18 FA	Brazil	MG	ON022249|Brazil|MG|Human|2018-01-16
ON022250	2018-01-16	Human	0136/18 FA_vaccinated	Brazil	MG	ON022250|Brazil|MG|Human|2018-01-16
ON022251	2018-01-05	Monkey	0048/18 IV	Brazil	MG	ON022251|Brazil|MG|Monkey|2018-01-05
ON022252	2018-01-01	Monkey	0047/18 IV	Brazil	MG	ON022252|Brazil|MG|Monkey|2018-01-01
ON022253	2018-01-17	Human	0163/18 FA_vaccinated	Brazil	MG	ON022253|Brazil|MG|Human|2018-01-17
ON022254	2018-01-04	Monkey	0045/18 IV	Brazil	MG	ON022254|Brazil|MG|Monkey|2018-01-04
ON022255	2018-01-15	Human	0092/18 FA	Brazil	MG	ON022255|Brazil|MG|Human|2018-01-15
ON022256	2018-01-13	Human	0091/18 FA	Brazil	MG	ON022256|Brazil|MG|Human|2018-01-13
ON022257	2018-01-08	Human	0025/18 FA	Brazil	MG	ON022257|Brazil|MG|Human|2018-01-08
ON022258	2017-12-30	Monkey	0043/18 IV	Brazil	MG	ON022258|Brazil|MG|Monkey|2017-12-30
ON022259	2018-01-02	Monkey	0049/18 IV	Brazil	MG	ON022259|Brazil|MG|Monkey|2018-01-02
ON022260	2018-01-14	Human	0068/18 FA	Brazil	MG	ON022260|Brazil|MG|Human|2018-01-14
ON022262	2018-01-06	Monkey	0046/18 IV	Brazil	MG	ON022262|Brazil|MG|Monkey|2018-01-06
ON022263	2018-01-10	Human	0043/18 FA	Brazil	MG	ON022263|Brazil|MG|Human|2018-01-10
ON022264	2017-12-18	Monkey	0007/18 IV	Brazil	MG	ON022264|Brazil|MG|Monkey|2017-12-18
ON022265	2018-01-04	Monkey	0055/18 IV	Brazil	MG	ON022265|Brazil|MG|Monkey|2018-01-04
ON022266	2017-12-26	Monkey	0005/18 IV	Brazil	MG	ON022266|Brazil|MG|Monkey|2017-12-26
ON022267	2018-01-15	Human	0109/18 FA	Brazil	MG	ON022267|Brazil|MG|Human|2018-01-15

ON022268	2018-01-16	Human	0299/2018		Brazil	RJ	ON022268|Brazil|RJ|Human|2018-01-16
ON022269	2017-11-28	Monkey	7922/2017		Brazil	RJ	ON022269|Brazil|RJ|Monkey|2017-11-28
ON022270	2018-01-17	Human	0276/2018		Brazil	RJ	ON022270|Brazil|RJ|Human|2018-01-17
ON022271	2018-01-16	Human	0331/2018		Brazil	RJ	ON022271|Brazil|RJ|Human|2018-01-16
ON022273	2018-01-13	Human	0227/2018		Brazil	RJ	ON022273|Brazil|RJ|Human|2018-01-13
ON022274	2018-01-13	Human	0229/2018		Brazil	RJ	ON022274|Brazil|RJ|Human|2018-01-13
ON022275	2018-01-14	Human	0232/2018		Brazil	RJ	ON022275|Brazil|RJ|Human|2018-01-14
ON022276	2018-01-15	Human	0303/2018		Brazil	RJ	ON022276|Brazil|RJ|Human|2018-01-15
ON022277	2018-01-15	Human	0306/2018		Brazil	RJ	ON022277|Brazil|RJ|Human|2018-01-15
ON022278	2018-01-15	Human	0318/2018		Brazil	RJ	ON022278|Brazil|RJ|Human|2018-01-15
ON022280	2017-12-15	Monkey	0023/2018		Brazil	RJ	ON022280|Brazil|RJ|Monkey|2017-12-15
ON022281	2017-12-22	Monkey	0101/2018		Brazil	RJ	ON022281|Brazil|RJ|Monkey|2017-12-22
ON022282	2018-01-23	Human	TEC1053		Brazil	RJ	ON022282|Brazil|RJ|Human|2018-01-23
ON022283	2018-11-21	Monkey		4965	Brazil	SP	ON022283|Brazil|SP|Monkey|2018-11-21
ON022284	2018-01-11	Monkey		4950	Brazil	SP	ON022284|Brazil|SP|Monkey|2018-01-11
ON022285	2018-03-11	Monkey		3383	Brazil	SP	ON022285|Brazil|SP|Monkey|2018-03-11
ON022286	2018-06-25	Mosquito		3766	Brazil	SP	ON022286|Brazil|SP|Mosquito|2018-06-25
ON022287	2018-07-20	Monkey		4393	Brazil	SP	ON022287|Brazil|SP|Monkey|2018-07-20
ON022288	2016-11-16	Monkey	IAL-10		Brazil	SP	ON022288|Brazil|SP|Monkey|2016-11-16
ON022289	2018-05-23	Mosquito		3551	Brazil	SP	ON022289|Brazil|SP|Mosquito|2018-05-23
ON022290	2018-05-23	Mosquito		3552	Brazil	SP	ON022290|Brazil|SP|Mosquito|2018-05-23
ON022291	2018-05-28	Mosquito		3541	Brazil	SP	ON022291|Brazil|SP|Mosquito|2018-05-28
ON022292	2018-03-20	Monkey		3551	Brazil	SP	ON022292|Brazil|SP|Monkey|2018-03-20
ON022293	2018-05-28	Mosquito		3530	Brazil	SP	ON022293|Brazil|SP|Mosquito|2018-05-28
ON022294	2018-05-28	Mosquito		3521	Brazil	SP	ON022294|Brazil|SP|Mosquito|2018-05-28
ON022295	2018-04-23	Mosquito		2377	Brazil	SP	ON022295|Brazil|SP|Mosquito|2018-04-23
ON022296	2018-03-02	Monkey		3255	Brazil	SP	ON022296|Brazil|SP|Monkey|2018-03-02
ON022297	2018-01-18	Monkey		4956	Brazil	SP	ON022297|Brazil|SP|Monkey|2018-01-18
ON022298	2018-03-23	Human		36384	Brazil	SP	ON022298|Brazil|SP|Human|2018-03-23
ON022299	2018-03-12	Monkey		4976	Brazil	SP	ON022299|Brazil|SP|Monkey|2018-03-12
ON022300	2016-11-03	Mosquito	IAL-1		Brazil	SP	ON022300|Brazil|SP|Mosquito|2016-11-03
ON022301	2016-11-03	Mosquito	IAL-2		Brazil	SP	ON022301|Brazil|SP|Mosquito|2016-11-03
ON022302	2016-11-03	Mosquito	IAL-3		Brazil	SP	ON022302|Brazil|SP|Mosquito|2016-11-03
ON022303	2017-01-24	Monkey	IAL-6		Brazil	SP	ON022303|Brazil|SP|Monkey|2017-01-24

ON022304	2018-07-04	Mosquito		3687	Brazil	SP	ON022304|Brazil|SP|Mosquito|2018-07-04
ON022305	2018-07-04	Mosquito		3689	Brazil	SP	ON022305|Brazil|SP|Mosquito|2018-07-04
ON022306	2018-05-13	Monkey		4086	Brazil	SP	ON022306|Brazil|SP|Monkey|2018-05-13
ON022307	2018-04-20	Human		37071	Brazil	SP	ON022307|Brazil|SP|Human|2018-04-20
ON022308	2018-04-02	Monkey		3587	Brazil	SP	ON022308|Brazil|SP|Monkey|2018-04-02
ON022309	2018-04-11	Monkey		3769	Brazil	SP	ON022309|Brazil|SP|Monkey|2018-04-11
ON022310	2018-03-10	Human		35903	Brazil	SP	ON022310|Brazil|SP|Human|2018-03-10
ON022311	2016-12-19	Monkey	IAL-14		Brazil	SP	ON022311|Brazil|SP|Monkey|2016-12-19
ON022312	2018-03-24	Monkey		3531	Brazil	SP	ON022312|Brazil|SP|Monkey|2018-03-24
ON022313	2018-03-24	Monkey		3538	Brazil	SP	ON022313|Brazil|SP|Monkey|2018-03-24
ON022314	2018-04-16	Monkey		4869	Brazil	SP	ON022314|Brazil|SP|Monkey|2018-04-16
ON022315	2018-03-21	Human	TEC1622		Brazil	SP	ON022315|Brazil|SP|Human|2018-03-21
ON022317	2018-03-12	Human		1086	Brazil	SP	ON022317|Brazil|SP|Human|2018-03-12
ON022318	2018-03-05	Human		1079	Brazil	SP	ON022318|Brazil|SP|Human|2018-03-05
ON022319	2019-01-XX	Human	SOFFA		Brazil	SP	ON022319|Brazil|SP|Human|2019-01-XX
ON022320	2018-01-08	Monkey		2864	Brazil	SP	ON022320|Brazil|SP|Monkey|2018-01-08
ON022321	2017-12-29	Monkey		2878	Brazil	SP	ON022321|Brazil|SP|Monkey|2017-12-29
ON022322	2018-01-07	Monkey		2885	Brazil	SP	ON022322|Brazil|SP|Monkey|2018-01-07
ON022323	2018-01-10	Monkey		2918	Brazil	SP	ON022323|Brazil|SP|Monkey|2018-01-10
ON022324	2018-01-22	Monkey		2965	Brazil	SP	ON022324|Brazil|SP|Monkey|2018-01-22
ON022325	2018-01-21	Monkey		2966	Brazil	SP	ON022325|Brazil|SP|Monkey|2018-01-21
ON022326	2018-01-19	Monkey		2967	Brazil	SP	ON022326|Brazil|SP|Monkey|2018-01-19
ON022327	2018-01-31	Monkey		3036	Brazil	SP	ON022327|Brazil|SP|Monkey|2018-01-31
ON022328	2018-02-22	Monkey		3217	Brazil	SP	ON022328|Brazil|SP|Monkey|2018-02-22
ON022329	2018-01-16	Monkey		2968	Brazil	SP	ON022329|Brazil|SP|Monkey|2018-01-16
ON022330	2018-01-19	Monkey		2971	Brazil	SP	ON022330|Brazil|SP|Monkey|2018-01-19
ON022331	2018-01-19	Monkey		2972	Brazil	SP	ON022331|Brazil|SP|Monkey|2018-01-19
ON022332	2018-01-19	Monkey		2973	Brazil	SP	ON022332|Brazil|SP|Monkey|2018-01-19
ON022333	2018-01-29	Monkey		3006	Brazil	SP	ON022333|Brazil|SP|Monkey|2018-01-29
ON022334	2018-01-31	Monkey		3035	Brazil	SP	ON022334|Brazil|SP|Monkey|2018-01-31
ON022335	2018-01-31	Monkey		3037	Brazil	SP	ON022335|Brazil|SP|Monkey|2018-01-31
ON022336	2018-01-29	Monkey		3038	Brazil	SP	ON022336|Brazil|SP|Monkey|2018-01-29
ON022337	2018-02-10	Monkey		3172	Brazil	SP	ON022337|Brazil|SP|Monkey|2018-02-10
ON022338	2018-01-21	Monkey		3211	Brazil	SP	ON022338|Brazil|SP|Monkey|2018-01-21

ON022339	2018-02-19	Monkey		3214	Brazil	SP	ON022339|Brazil|SP|Monkey|2018-02-19
ON022340	2018-02-15	Monkey		3216	Brazil	SP	ON022340|Brazil|SP|Monkey|2018-02-15
ON022341	2018-01-02	Monkey		2866	Brazil	SP	ON022341|Brazil|SP|Monkey|2018-01-02
ON022342	2018-01-30	Monkey		3199	Brazil	SP	ON022342|Brazil|SP|Monkey|2018-01-30
ON022343	2018-01-15	Monkey		3202	Brazil	SP	ON022343|Brazil|SP|Monkey|2018-01-15
ON022344	2018-02-01	Monkey		3223	Brazil	SP	ON022344|Brazil|SP|Monkey|2018-02-01
ON022345	2018-03-01	Monkey		3238	Brazil	SP	ON022345|Brazil|SP|Monkey|2018-03-01
ON022346	2018-01-26	Monkey		3240	Brazil	SP	ON022346|Brazil|SP|Monkey|2018-01-26
ON022347	2018-03-03	Monkey		3266	Brazil	SP	ON022347|Brazil|SP|Monkey|2018-03-03
ON022348	2018-03-13	Monkey		3382	Brazil	SP	ON022348|Brazil|SP|Monkey|2018-03-13
ON022349	2018-03-10	Monkey		3450	Brazil	SP	ON022349|Brazil|SP|Monkey|2018-03-10
ON022350	2018-03-14	Monkey		3462	Brazil	SP	ON022350|Brazil|SP|Monkey|2018-03-14
ON022351	2018-03-27	Monkey		3563	Brazil	SP	ON022351|Brazil|SP|Monkey|2018-03-27
ON022352	2018-04-18	Monkey		3773	Brazil	SP	ON022352|Brazil|SP|Monkey|2018-04-18
ON022353	2017-12-27	Monkey		2752	Brazil	SP	ON022353|Brazil|SP|Monkey|2017-12-27
ON022354	2017-12-27	Monkey		2755	Brazil	SP	ON022354|Brazil|SP|Monkey|2017-12-27
ON022355	2017-12-27	Monkey		2756	Brazil	SP	ON022355|Brazil|SP|Monkey|2017-12-27
ON022356	2017-12-27	Monkey		2757	Brazil	SP	ON022356|Brazil|SP|Monkey|2017-12-27
ON022357	2017-12-27	Monkey		2782	Brazil	SP	ON022357|Brazil|SP|Monkey|2017-12-27
ON022358	2018-01-03	Monkey		2783	Brazil	SP	ON022358|Brazil|SP|Monkey|2018-01-03
ON022359	2017-12-26	Monkey		2786	Brazil	SP	ON022359|Brazil|SP|Monkey|2017-12-26
ON022360	2018-01-02	Monkey		2868	Brazil	SP	ON022360|Brazil|SP|Monkey|2018-01-02
ON022361	2018-01-05	Monkey		2869	Brazil	SP	ON022361|Brazil|SP|Monkey|2018-01-05
ON022362	2017-12-26	Monkey		2788	Brazil	SP	ON022362|Brazil|SP|Monkey|2017-12-26
ON022363	2017-12-26	Monkey		2789	Brazil	SP	ON022363|Brazil|SP|Monkey|2017-12-26
ON022364	2017-12-29	Monkey		2862	Brazil	SP	ON022364|Brazil|SP|Monkey|2017-12-29
ON022365	2018-01-05	Monkey		2870	Brazil	SP	ON022365|Brazil|SP|Monkey|2018-01-05
ON022366	2018-01-05	Monkey		2872	Brazil	SP	ON022366|Brazil|SP|Monkey|2018-01-05
ON022367	2019-01-21	Monkey		5148	Brazil	SP	ON022367|Brazil|SP|Monkey|2019-01-21
ON022368	2019-01-15	Monkey		5159	Brazil	SP	ON022368|Brazil|SP|Monkey|2019-01-15
ON022369	2019-01-31	Monkey		5194	Brazil	SP	ON022369|Brazil|SP|Monkey|2019-01-31
ON022370	2019-02-19	Monkey		5312	Brazil		ON022370|Brazil||Monkey|2019-02-19
ON022371	2017-07-03	Monkey	1594/17 IV		Brazil	MG	ON022371|Brazil|MG|Monkey|2017-07-03
ON022372	2017-11-17	Monkey	2014/17 IV		Brazil	MG	ON022372|Brazil|MG|Monkey|2017-11-17

ON022373	2017-11-18	Monkey	1969/17 IV	Brazil	MG	ON022373|Brazil|MG|Monkey|2017-11-18
ON022374	2017-03-13	Monkey	0881/17 IV	Brazil	MG	ON022374|Brazil|MG|Monkey|2017-03-13
ON022375	2017-02-12	Monkey	0407/17 IV	Brazil	MG	ON022375|Brazil|MG|Monkey|2017-02-12
ON022376	2018-04-11	Monkey	0891/18 IV	Brazil	MG	ON022376|Brazil|MG|Monkey|2018-04-11
ON022377	2018-04-17	Monkey	0934/18 IV	Brazil	MG	ON022377|Brazil|MG|Monkey|2018-04-17
ON022378	2017-11-09	Monkey	1937/17 IV	Brazil	MG	ON022378|Brazil|MG|Monkey|2017-11-09
ON022379	2017-05-17	Monkey	1351/17 IV	Brazil	MG	ON022379|Brazil|MG|Monkey|2017-05-17
ON022380	2017-11-16	Monkey	1943/17 IV	Brazil	MG	ON022380|Brazil|MG|Monkey|2017-11-16
ON022381	2017-04-06	Monkey	0955/17 IV	Brazil	MG	ON022381|Brazil|MG|Monkey|2017-04-06
ON022382	2017-07-03	Monkey	1597/17 IV	Brazil	MG	ON022382|Brazil|MG|Monkey|2017-07-03
ON022383	2017-04-03	Monkey	0843/17 IV	Brazil	MG	ON022383|Brazil|MG|Monkey|2017-04-03
ON022384	2017-04-22	Monkey	1205/17 IV	Brazil	MG	ON022384|Brazil|MG|Monkey|2017-04-22
ON022385	2017-11-24	Monkey	1974/17 IV	Brazil	MG	ON022385|Brazil|MG|Monkey|2017-11-24
ON022386	2017-04-18	Monkey	1192/17 IV	Brazil	MG	ON022386|Brazil|MG|Monkey|2017-04-18
ON022387	2017-12-04	Monkey	2043/17 IV	Brazil	MG	ON022387|Brazil|MG|Monkey|2017-12-04
ON022388	2017-03-13	Monkey	0883/17 IV	Brazil	MG	ON022388|Brazil|MG|Monkey|2017-03-13
ON022389	2017-04-12	Monkey	1040/17 IV	Brazil	MG	ON022389|Brazil|MG|Monkey|2017-04-12
ON022390	2017-04-06	Monkey	0954/17 IV	Brazil	MG	ON022390|Brazil|MG|Monkey|2017-04-06
ON022391	2017-11-25	Monkey	2009/17 IV	Brazil	MG	ON022391|Brazil|MG|Monkey|2017-11-25
ON022392	2018-01-29	Monkey	0314/18 IV	Brazil	MG	ON022392|Brazil|MG|Monkey|2018-01-29
ON022393	2017-12-05	Monkey	2033/17 IV	Brazil	MG	ON022393|Brazil|MG|Monkey|2017-12-05
ON022394	2018-04-06	Monkey	0884/18 IV	Brazil	MG	ON022394|Brazil|MG|Monkey|2018-04-06
ON022395	2017-03-24	Monkey	0817/17 IV	Brazil	MG	ON022395|Brazil|MG|Monkey|2017-03-24
ON022396	2017-03-23	Monkey	0929/17 IV	Brazil	MG	ON022396|Brazil|MG|Monkey|2017-03-23
ON022397	2017-11-20	Monkey	1973/17 IV	Brazil	MG	ON022397|Brazil|MG|Monkey|2017-11-20
ON022398	2017-07-17	Monkey	1689/17 IV	Brazil	MG	ON022398|Brazil|MG|Monkey|2017-07-17
ON022399	2017-03-14	Monkey	0872/17 IV	Brazil	MG	ON022399|Brazil|MG|Monkey|2017-03-14
ON022401	2017-03-28	Monkey	0899/17 IV	Brazil	MG	ON022401|Brazil|MG|Monkey|2017-03-28
ON022402	2017-11-07	Monkey	1908/17 IV	Brazil	MG	ON022402|Brazil|MG|Monkey|2017-11-07
ON022403	2017-11-07	Monkey	1967/17 IV	Brazil	MG	ON022403|Brazil|MG|Monkey|2017-11-07
ON022404	2017-11-30	Monkey	2000/17 IV	Brazil	MG	ON022404|Brazil|MG|Monkey|2017-11-30
ON022405	2017-10-27	Monkey	1912/17 IV	Brazil	MG	ON022405|Brazil|MG|Monkey|2017-10-27
ON022406	2017-10-25	Monkey	1965/17 IV	Brazil	MG	ON022406|Brazil|MG|Monkey|2017-10-25
ON022407	2017-03-13	Monkey	0869/17 IV	Brazil	MG	ON022407|Brazil|MG|Monkey|2017-03-13

ON022408	2017-05-04	Monkey	1309/17 IV	Brazil	MG	ON022408|Brazil|MG|Monkey|2017-05-04
ON022409	2017-04-18	Monkey	1209/17 IV	Brazil	MG	ON022409|Brazil|MG|Monkey|2017-04-18
ON022410	2017-04-06	Monkey	0949/17 IV	Brazil	MG	ON022410|Brazil|MG|Monkey|2017-04-06
ON022411	2017-12-05	Monkey	2002/17 IV	Brazil	MG	ON022411|Brazil|MG|Monkey|2017-12-05
ON022412	2017-07-04	Monkey	1593/17 IV	Brazil	MG	ON022412|Brazil|MG|Monkey|2017-07-04
ON022413	2017-11-08	Monkey	1942/17 IV	Brazil	MG	ON022413|Brazil|MG|Monkey|2017-11-08
ON022414	2017-04-03	Monkey	0952/17 IV	Brazil	MG	ON022414|Brazil|MG|Monkey|2017-04-03
ON022415	2017-04-03	Monkey	0849/17 IV	Brazil	MG	ON022415|Brazil|MG|Monkey|2017-04-03
ON022416	2017-05-05	Monkey	1258/17 IV	Brazil	MG	ON022416|Brazil|MG|Monkey|2017-05-05
ON022417	2017-10-31	Monkey	1938/17 IV	Brazil	MG	ON022417|Brazil|MG|Monkey|2017-10-31
ON022418	2017-03-24	Monkey	0810/17 IV	Brazil	MG	ON022418|Brazil|MG|Monkey|2017-03-24
ON022419	2017-11-05	Monkey	1902/17 IV	Brazil	MG	ON022419|Brazil|MG|Monkey|2017-11-05
ON022420	2017-12-13	Monkey	2077/17 IV	Brazil	MG	ON022420|Brazil|MG|Monkey|2017-12-13
ON022421	2017-04-11	Monkey	1204/17 IV	Brazil	MG	ON022421|Brazil|MG|Monkey|2017-04-11
ON022422	2017-03-25	Monkey	0927/17 IV	Brazil	MG	ON022422|Brazil|MG|Monkey|2017-03-25
ON022423	2017-11-29	Monkey	1975/17 IV	Brazil	MG	ON022423|Brazil|MG|Monkey|2017-11-29
ON022424	2017-12-21	Monkey	2081/17 IV	Brazil	MG	ON022424|Brazil|MG|Monkey|2017-12-21
ON022425	2017-10-26	Monkey	1911/17 IV	Brazil	MG	ON022425|Brazil|MG|Monkey|2017-10-26
ON022426	2017-03-14	Monkey	0877/17 IV	Brazil	MG	ON022426|Brazil|MG|Monkey|2017-03-14
ON022427	2017-12-03	Monkey	2042/17 IV	Brazil	MG	ON022427|Brazil|MG|Monkey|2017-12-03
ON022428	2017-03-02	Monkey	0614/17 IV	Brazil	MG	ON022428|Brazil|MG|Monkey|2017-03-02
ON022429	2017-04-18	Monkey	1193/17 IV	Brazil	MG	ON022429|Brazil|MG|Monkey|2017-04-18
ON022430	2017-03-21	Monkey	1015/17 IV	Brazil	MG	ON022430|Brazil|MG|Monkey|2017-03-21
ON022431	2017-12-05	Monkey	2005/17 IV	Brazil	MG	ON022431|Brazil|MG|Monkey|2017-12-05
ON022432	2017-12-07	Monkey	2065/17 IV	Brazil	MG	ON022432|Brazil|MG|Monkey|2017-12-07
ON022433	2018-02-26	Monkey	3048/2018	Brazil	RJ	ON022433|Brazil|RJ|Monkey|2018-02-26
ON022434	2018-02-08	Monkey	2222/2018	Brazil	RJ	ON022434|Brazil|RJ|Monkey|2018-02-08
ON022435	2017-10-23	Monkey	7578/2017	Brazil	RJ	ON022435|Brazil|RJ|Monkey|2017-10-23
ON022436	2018-02-26	Monkey	3052/2018	Brazil	RJ	ON022436|Brazil|RJ|Monkey|2018-02-26
ON022437	2018-02-05	Monkey	1542/2018	Brazil	RJ	ON022437|Brazil|RJ|Monkey|2018-02-05
ON022438	2018-03-20	Monkey	3953/2018	Brazil	RJ	ON022438|Brazil|RJ|Monkey|2018-03-20
ON022439	2017-04-20	Monkey	4884/2017	Brazil	RJ	ON022439|Brazil|RJ|Monkey|2017-04-20
ON022440	2018-02-05	Monkey	1544/2018	Brazil	RJ	ON022440|Brazil|RJ|Monkey|2018-02-05
ON022441	2018-04-09	Monkey	4789/2018	Brazil	RJ	ON022441|Brazil|RJ|Monkey|2018-04-09

ON022442	2018-04-17	Monkey	5509/2018	Brazil	RJ	ON022442|Brazil|RJ|Monkey|2018-04-17
ON022443	2017-05-19	Monkey	5597/2017	Brazil	RJ	ON022443|Brazil|RJ|Monkey|2017-05-19
ON022444	2017-03-20	Monkey	2142/2017	Brazil	ES	ON022444|Brazil|ES|Monkey|2017-03-20
ON022445	2018-01-22	Monkey	0791/2018	Brazil	RJ	ON022445|Brazil|RJ|Monkey|2018-01-22
ON022446	2017-04-03	Monkey	3058/2017	Brazil	ES	ON022446|Brazil|ES|Monkey|2017-04-03
ON022447	2017-04-02	Human	3480/2017	Brazil	ES	ON022447|Brazil|ES|Human|2017-04-02
ON022448	2018-04-12	Monkey	5058/2018	Brazil	RJ	ON022448|Brazil|RJ|Monkey|2018-04-12
ON022449	2018-03-13	Monkey	3637/2018	Brazil	RJ	ON022449|Brazil|RJ|Monkey|2018-03-13
ON022450	2018-01-24	Monkey	1036/2018	Brazil	RJ	ON022450|Brazil|RJ|Monkey|2018-01-24
ON022451	2018-01-18	Monkey	0803/2018	Brazil	RJ	ON022451|Brazil|RJ|Monkey|2018-01-18
ON022452	2018-02-19	Monkey	2454/2018	Brazil	RJ	ON022452|Brazil|RJ|Monkey|2018-02-19
ON022453	2018-02-28	Monkey	3144/2018	Brazil	RJ	ON022453|Brazil|RJ|Monkey|2018-02-28
ON022454	2018-02-19	Monkey	2380/2018	Brazil	RJ	ON022454|Brazil|RJ|Monkey|2018-02-19
ON022455	2017-05-02	Monkey	5280/2017	Brazil	RJ	ON022455|Brazil|RJ|Monkey|2017-05-02
ON022456	2017-01-23	Monkey	0250/2017	Brazil	ES	ON022456|Brazil|ES|Monkey|2017-01-23
ON022457	2018-04-03	Monkey	4580/2018	Brazil	RJ	ON022457|Brazil|RJ|Monkey|2018-04-03
ON022458	2018-02-05	Monkey	1537/2018	Brazil	RJ	ON022458|Brazil|RJ|Monkey|2018-02-05
ON022459	2018-03-13	Monkey	3634/2018	Brazil	RJ	ON022459|Brazil|RJ|Monkey|2018-03-13
ON022460	2017-04-17	Monkey	4271/2017	Brazil	RJ	ON022460|Brazil|RJ|Monkey|2017-04-17
ON022461	2017-01-24	Monkey	0253/2017	Brazil	ES	ON022461|Brazil|ES|Monkey|2017-01-24
ON022462	2018-04-13	Monkey	5046/2018	Brazil	RJ	ON022462|Brazil|RJ|Monkey|2018-04-13
ON022463	2017-04-17	Monkey	4301/2017	Brazil	RJ	ON022463|Brazil|RJ|Monkey|2017-04-17
ON022464	2017-01-26	Monkey	0440/2017	Brazil	ES	ON022464|Brazil|ES|Monkey|2017-01-26
ON022465	2018-04-03	Monkey	4584/2018	Brazil	RJ	ON022465|Brazil|RJ|Monkey|2018-04-03
ON022466	2017-05-19	Monkey	5599/2017	Brazil	RJ	ON022466|Brazil|RJ|Monkey|2017-05-19
ON022467	2017-04-24	Monkey	4886/2017	Brazil	RJ	ON022467|Brazil|RJ|Monkey|2017-04-24
ON022468	2017-03-06	Monkey	2123/2017	Brazil	ES	ON022468|Brazil|ES|Monkey|2017-03-06
ON022469	2017-04-20	Monkey	4878/2017	Brazil	RJ	ON022469|Brazil|RJ|Monkey|2017-04-20
ON022470	2018-02-15	Human	1934/2018	Brazil	RJ	ON022470|Brazil|RJ|Human|2018-02-15
ON022471	2018-01-24	Human	0612/2018	Brazil	RJ	ON022471|Brazil|RJ|Human|2018-01-24
ON022472	2018-02-24	Human	2643/2018	Brazil	RJ	ON022472|Brazil|RJ|Human|2018-02-24
ON022473	2018-02-16	Human	2291/2018	Brazil	RJ	ON022473|Brazil|RJ|Human|2018-02-16
ON022474	2018-02-18	Human	5435/2018	Brazil	MG	ON022474|Brazil|MG|Human|2018-02-18
ON022475	2018-02-22	Human	2544/2018	Brazil	RJ	ON022475|Brazil|RJ|Human|2018-02-22

ON022476	2018-02-06	Human	1617/2018	Brazil	RJ	ON022476|Brazil|RJ|Human|2018-02-06
ON022477	2018-02-14	Human	1949/2018	Brazil	RJ	ON022477|Brazil|RJ|Human|2018-02-14
ON022478	2018-02-09	Human	2054/2018	Brazil	RJ	ON022478|Brazil|RJ|Human|2018-02-09
ON022479	2018-01-24	Human	0730/2018	Brazil	RJ	ON022479|Brazil|RJ|Human|2018-01-24
ON022480	2018-02-19	Human	2301/2018	Brazil	RJ	ON022480|Brazil|RJ|Human|2018-02-19
ON022481	2018-02-16	Human	2055/2018	Brazil	RJ	ON022481|Brazil|RJ|Human|2018-02-16
ON022482	2018-02-20	Human	2540/2018	Brazil	RJ	ON022482|Brazil|RJ|Human|2018-02-20
ON022483	2018-02-16	Human	2138/2018	Brazil	RJ	ON022483|Brazil|RJ|Human|2018-02-16
ON022484	2018-02-20	Human	2137/2018	Brazil	RJ	ON022484|Brazil|RJ|Human|2018-02-20
ON022485	2018-02-08	Human	1781/2018	Brazil	RJ	ON022485|Brazil|RJ|Human|2018-02-08
ON022486	2018-02-22	Human	2634/2018	Brazil	RJ	ON022486|Brazil|RJ|Human|2018-02-22
ON022487	2018-02-19	Human	2136/2018	Brazil	RJ	ON022487|Brazil|RJ|Human|2018-02-19
ON022488	2018-02-11	Human	1828/2018	Brazil	RJ	ON022488|Brazil|RJ|Human|2018-02-11
ON022489	2018-02-06	Human	1797/2018	Brazil	RJ	ON022489|Brazil|RJ|Human|2018-02-06
ON022490	2018-02-15	Human	1950/2018	Brazil	RJ	ON022490|Brazil|RJ|Human|2018-02-15
ON022491	2018-02-09	Human	2153/2018	Brazil	RJ	ON022491|Brazil|RJ|Human|2018-02-09
ON022492	2018-01-20	Human	0569/2018	Brazil	RJ	ON022492|Brazil|RJ|Human|2018-01-20
ON022493	2018-02-21	Human	2498/2018	Brazil	RJ	ON022493|Brazil|RJ|Human|2018-02-21
ON022494	2018-02-17	Human	2663/2018	Brazil	RJ	ON022494|Brazil|RJ|Human|2018-02-17
ON022495	2018-02-09	Human	2334/2018	Brazil	RS	ON022495|Brazil|RS|Human|2018-02-09
ON022496	2017-02-24	Human	1547/2017	Brazil	ES	ON022496|Brazil|ES|Human|2017-02-24
ON022497	2018-02-21	Human	2545/2018	Brazil	RJ	ON022497|Brazil|RJ|Human|2018-02-21
ON022498	2018-01-25	Human	0913/2018	Brazil	RJ	ON022498|Brazil|RJ|Human|2018-01-25
ON022499	2018-02-21	Human	2497/2018	Brazil	RJ	ON022499|Brazil|RJ|Human|2018-02-21
ON022500	2018-02-19	Human	2176/2018	Brazil	RJ	ON022500|Brazil|RJ|Human|2018-02-19
ON022501	2018-02-20	Human	2146/2018	Brazil	RJ	ON022501|Brazil|RJ|Human|2018-02-20
ON022502	2018-02-19	Human	2139/2018	Brazil	RJ	ON022502|Brazil|RJ|Human|2018-02-19
ON022503	2018-02-05	Human	1840/2018	Brazil	RJ	ON022503|Brazil|RJ|Human|2018-02-05
ON022504	2017-03-17	Human	2153/2017	Brazil	ES	ON022504|Brazil|ES|Human|2017-03-17
ON022505	2018-02-21	Human	2051/2018	Brazil	RJ	ON022505|Brazil|RJ|Human|2018-02-21
ON022506	2018-01-24	Human	1004/2018	Brazil	RJ	ON022506|Brazil|RJ|Human|2018-01-24
ON022507	2018-01-18	Human	0568/2018	Brazil	RJ	ON022507|Brazil|RJ|Human|2018-01-18
ON022508	2018-01-25	Human	0921/2018	Brazil	RJ	ON022508|Brazil|RJ|Human|2018-01-25
ON022509	2018-02-05	Human	1577/2018	Brazil	RJ	ON022509|Brazil|RJ|Human|2018-02-05

ON022510	2017-03-02	Human	1532/2017	Brazil	ES	ON022510|Brazil|ES|Human|2017-03-02
ON022511	2018-02-22	Human	2555/2018	Brazil	RJ	ON022511|Brazil|RJ|Human|2018-02-22
ON022512	2018-02-20	Human	2551/2018	Brazil	RJ	ON022512|Brazil|RJ|Human|2018-02-20
ON022513	2018-02-11	Human	2151/2018	Brazil	RJ	ON022513|Brazil|RJ|Human|2018-02-11
ON022514	2018-01-24	Human	0620/2018	Brazil	RJ	ON022514|Brazil|RJ|Human|2018-01-24
ON022515	2018-02-22	Human	2635/2018	Brazil	RJ	ON022515|Brazil|RJ|Human|2018-02-22
ON022516	2018-01-20	Human	0402/2018	Brazil	RJ	ON022516|Brazil|RJ|Human|2018-01-20
ON022517	2018-02-05	Monkey	1868/2018	Brazil	RJ	ON022517|Brazil|RJ|Monkey|2018-02-05
ON022518	2017-02-27	Monkey	2069/2017	Brazil	ES	ON022518|Brazil|ES|Monkey|2017-02-27
ON022519	2017-04-18	Monkey	4871/2017	Brazil	RJ	ON022519|Brazil|RJ|Monkey|2017-04-18
ON022520	2017-03-07	Human	1813/2017	Brazil	ES	ON022520|Brazil|ES|Human|2017-03-07
ON022521	2017-04-25	Human	5022/2017	Brazil	RJ	ON022521|Brazil|RJ|Human|2017-04-25
ON022522	2017-06-02	Human	5817/2017	Brazil	RJ	ON022522|Brazil|RJ|Human|2017-06-02
ON022523	2017-02-21	Human	1442/2017	Brazil	ES	ON022523|Brazil|ES|Human|2017-02-21
ON022524	2017-01-30	Human	0712/2017	Brazil	ES	ON022524|Brazil|ES|Human|2017-01-30
ON022525	2017-XX-XX	Human	1526/2017	Brazil	ES	ON022525|Brazil|ES|Human|2017-XX-XX
ON022526	2018-02-13	Human	1839/2018	Brazil	RJ	ON022526|Brazil|RJ|Human|2018-02-13
ON022527	2017-02-22	Human	1522/2017	Brazil	ES	ON022527|Brazil|ES|Human|2017-02-22
ON022528	2017-02-12	Human	1527/2017	Brazil	ES	ON022528|Brazil|ES|Human|2017-02-12
ON022529	2017-03-16	Human	1994/2017	Brazil	ES	ON022529|Brazil|ES|Human|2017-03-16
ON022530	2018-01-25	Monkey	3851/2018	Brazil	ES	ON022530|Brazil|ES|Monkey|2018-01-25
ON022531	2017-01-20	Human	0231/2017	Brazil	ES	ON022531|Brazil|ES|Human|2017-01-20
ON022532	2017-01-29	Human	0404/2017	Brazil	ES	ON022532|Brazil|ES|Human|2017-01-29
ON022533	2018-02-11	Human	1821/2018	Brazil	RJ	ON022533|Brazil|RJ|Human|2018-02-11
ON022534	2018-02-27	Human	0766/2018	Brazil	RJ	ON022534|Brazil|RJ|Human|2018-02-27
ON022535	2017-01-26	Human	0277/2017	Brazil	ES	ON022535|Brazil|ES|Human|2017-01-26
ON022536	2017-01-25	Human	0273/2017	Brazil	ES	ON022536|Brazil|ES|Human|2017-01-25
ON022537	2017-02-06	Human	0760/2017	Brazil	ES	ON022537|Brazil|ES|Human|2017-02-06
ON022538	2017-01-27	Human	0725/2017	Brazil	ES	ON022538|Brazil|ES|Human|2017-01-27
ON022539	2017-02-10	Human	1123/2017	Brazil	ES	ON022539|Brazil|ES|Human|2017-02-10
ON022540	2018-03-11	Human	CP6	Brazil	SP	ON022540|Brazil|SP|Human|2018-03-11
ON022541	2018-03-28	Human	CP12	Brazil	SP	ON022541|Brazil|SP|Human|2018-03-28
ON022542	2018-03-28	Human	CP13	Brazil	SP	ON022542|Brazil|SP|Human|2018-03-28
ON022543	2018-04-11	Human	CP16	Brazil	SP	ON022543|Brazil|SP|Human|2018-04-11

ON022544	2018-04-15	Human	CP18	Brazil	SP	ON022544|Brazil|SP|Human|2018-04-15
ON022545	2019-01-15	Human	CP20	Brazil	SP	ON022545|Brazil|SP|Human|2019-01-15
ON022546	2019-01-15	Human	CP21	Brazil	SP	ON022546|Brazil|SP|Human|2019-01-15
ON022547	2019-01-18	Human	CP22	Brazil	SP	ON022547|Brazil|SP|Human|2019-01-18
ON022548	2019-01-19	Human	CP23	Brazil	SP	ON022548|Brazil|SP|Human|2019-01-19
ON022550	2019-01-30	Human	CP28	Brazil	SP	ON022550|Brazil|SP|Human|2019-01-30
ON022551	2019-02-17	Human	CP31	Brazil	SP	ON022551|Brazil|SP|Human|2019-02-17
ON022552	2019-02-24	Human	CP33	Brazil	SP	ON022552|Brazil|SP|Human|2019-02-24
ON022554	2018-03-14	Human	CP38	Brazil	SP	ON022554|Brazil|SP|Human|2018-03-14
ON022555	2019-01-09	Human	CP42	Brazil	SP	ON022555|Brazil|SP|Human|2019-01-09
ON022556	2019-01-14	Human	CP43	Brazil	SP	ON022556|Brazil|SP|Human|2019-01-14
ON022557	2019-01-14	Human	CP44	Brazil	SP	ON022557|Brazil|SP|Human|2019-01-14
ON022558	2019-01-20	Human	CP46	Brazil	SP	ON022558|Brazil|SP|Human|2019-01-20
ON022559	2019-01-31	Human	CP49	Brazil	SP	ON022559|Brazil|SP|Human|2019-01-31
ON022560	2019-02-06	Human	CP50	Brazil	SP	ON022560|Brazil|SP|Human|2019-02-06
ON022561	2019-02-07	Human	CP51	Brazil	SP	ON022561|Brazil|SP|Human|2019-02-07
ON022562	2019-02-07	Human	CP52	Brazil	SP	ON022562|Brazil|SP|Human|2019-02-07
ON022563	2019-01-20	Human	CP24	Brazil	SP	ON022563|Brazil|SP|Human|2019-01-20
ON022565	2018-01-02	Monkey	PNH2803	Brazil	SP	ON022565|Brazil|SP|Monkey|2018-01-02
ON022566	2017-12-21	Monkey	PNH2772	Brazil	SP	ON022566|Brazil|SP|Monkey|2017-12-21
ON022567	2017-12-21	Monkey	PNH2773	Brazil	SP	ON022567|Brazil|SP|Monkey|2017-12-21
ON022568	2017-12-28	Monkey	PNH2777	Brazil	SP	ON022568|Brazil|SP|Monkey|2017-12-28
ON022569	2018-01-06	Monkey	PNH2806	Brazil	SP	ON022569|Brazil|SP|Monkey|2018-01-06
ON022570	2017-12-19	Monkey	PNH2792	Brazil	SP	ON022570|Brazil|SP|Monkey|2017-12-19
ON022571	2017-12-20	Monkey	PNH2795	Brazil	SP	ON022571|Brazil|SP|Monkey|2017-12-20
ON022572	2018-03-01	Monkey	PNH3568	Brazil	SP	ON022572|Brazil|SP|Monkey|2018-03-01
ON022573	2017-03-28	Monkey	PNH951	Brazil	SP	ON022573|Brazil|SP|Monkey|2017-03-28
ON022574	2017-03-17	Monkey	PNH968	Brazil	SP	ON022574|Brazil|SP|Monkey|2017-03-17
ON022575	2017-03-27	Monkey	PNH880	Brazil	SP	ON022575|Brazil|SP|Monkey|2017-03-27
ON022576	2017-04-04	Monkey	PNH983	Brazil	SP	ON022576|Brazil|SP|Monkey|2017-04-04
ON022577	2018-06-08	Monkey	PNH4179/1	Brazil	SP	ON022577|Brazil|SP|Monkey|2018-06-08
ON022578	2017-02-24	Monkey	PNH558	Brazil	SP	ON022578|Brazil|SP|Monkey|2017-02-24
ON022579	2017-04-01	Monkey	PNH990	Brazil	SP	ON022579|Brazil|SP|Monkey|2017-04-01
ON022580	2017-04-03	Monkey	PNH987	Brazil	SP	ON022580|Brazil|SP|Monkey|2017-04-03

ON022581	2017-01-23	Monkey		302	Brazil	SP	ON022581|Brazil|SP|Monkey|2017-01-23
ON022582	2017-02-27	Monkey	PNH493		Brazil	SP	ON022582|Brazil|SP|Monkey|2017-02-27
ON022583	2018-06-05	Monkey	PNH4183		Brazil	SP	ON022583|Brazil|SP|Monkey|2018-06-05
ON022584	2018-03-17	Monkey	PNH3452		Brazil	SP	ON022584|Brazil|SP|Monkey|2018-03-17
ON022585	2018-03-15	Monkey	PNH3460		Brazil	SP	ON022585|Brazil|SP|Monkey|2018-03-15
ON022586	2018-01-14	Monkey	PNH2915		Brazil	SP	ON022586|Brazil|SP|Monkey|2018-01-14
ON022587	2018-01-28	Monkey	PNH2984		Brazil	SP	ON022587|Brazil|SP|Monkey|2018-01-28
ON022588	2018-01-22	Monkey	PNH2987		Brazil	SP	ON022588|Brazil|SP|Monkey|2018-01-22
ON022589	2018-01-31	Monkey	PNH3025		Brazil	SP	ON022589|Brazil|SP|Monkey|2018-01-31
ON022590	2018-01-31	Monkey	PNH3089		Brazil	SP	ON022590|Brazil|SP|Monkey|2018-01-31
ON022591	2018-02-09	Monkey	PNH3254		Brazil	SP	ON022591|Brazil|SP|Monkey|2018-02-09
ON022592	2018-02-19	Monkey	PNH3273		Brazil	SP	ON022592|Brazil|SP|Monkey|2018-02-19
ON022593	2018-03-21	Monkey	PNH3533		Brazil	SP	ON022593|Brazil|SP|Monkey|2018-03-21
ON022594	2018-01-23	Monkey	PNH2989		Brazil	SP	ON022594|Brazil|SP|Monkey|2018-01-23
ON022595	2018-05-03	Monkey	PNH3896		Brazil	SP	ON022595|Brazil|SP|Monkey|2018-05-03
ON022596	2017-04-29	Monkey	PNH1296		Brazil	SP	ON022596|Brazil|SP|Monkey|2017-04-29
ON022597	2018-01-16	Monkey	PNH3349		Brazil	SP	ON022597|Brazil|SP|Monkey|2018-01-16
ON022598	2018-01-17	Monkey	PNH3350		Brazil	SP	ON022598|Brazil|SP|Monkey|2018-01-17
ON022599	2018-02-20	Monkey	PNH3299		Brazil	SP	ON022599|Brazil|SP|Monkey|2018-02-20
ON022600	2016-12-13	Monkey	PNH152		Brazil	SP	ON022600|Brazil|SP|Monkey|2016-12-13
ON022601	2017-12-25	Monkey	PNH2754		Brazil	SP	ON022601|Brazil|SP|Monkey|2017-12-25
ON022602	2018-02-09	Monkey	PNH3278_duplicate		Brazil	SP	ON022602|Brazil|SP|Monkey|2018-02-09
ON022603	2018-01-04	Monkey	PNH2871		Brazil	SP	ON022603|Brazil|SP|Monkey|2018-01-04
ON022604	2017-06-04	Monkey	PNH1641		Brazil	SP	ON022604|Brazil|SP|Monkey|2017-06-04
ON022605	2017-06-14	Monkey	PNH1648		Brazil	SP	ON022605|Brazil|SP|Monkey|2017-06-14
ON022606	2017-07-04	Monkey	PNH1766		Brazil	SP	ON022606|Brazil|SP|Monkey|2017-07-04
ON022607	2017-07-07	Monkey	PNH1790		Brazil	SP	ON022607|Brazil|SP|Monkey|2017-07-07
ON022608	2017-05-05	Monkey	PNH1396		Brazil	SP	ON022608|Brazil|SP|Monkey|2017-05-05
ON022609	2017-12-08	Monkey	PNH2655		Brazil	SP	ON022609|Brazil|SP|Monkey|2017-12-08
ON022610	2017-07-17	Monkey	PNH1794		Brazil	SP	ON022610|Brazil|SP|Monkey|2017-07-17
ON022611	2017-04-17	Monkey	PNH1251		Brazil	SP	ON022611|Brazil|SP|Monkey|2017-04-17
ON022612	2017-04-17	Monkey	PNH1268		Brazil	SP	ON022612|Brazil|SP|Monkey|2017-04-17
ON022613	2017-05-17	Monkey	PNH1470		Brazil	SP	ON022613|Brazil|SP|Monkey|2017-05-17
ON022614	2017-05-11	Monkey	PNH1502		Brazil	SP	ON022614|Brazil|SP|Monkey|2017-05-11

ON022615	2017-04-10	Monkey	PNH1255	Brazil	SP	ON022615|Brazil|SP|Monkey|2017-04-10
ON022616	2017-04-20	Monkey	PNH1238	Brazil	SP	ON022616|Brazil|SP|Monkey|2017-04-20
ON022617	2017-05-03	Monkey	PNH1299	Brazil	SP	ON022617|Brazil|SP|Monkey|2017-05-03
ON022618	2017-04-16	Monkey	PNH1265	Brazil	SP	ON022618|Brazil|SP|Monkey|2017-04-16
ON022619	2017-04-28	Monkey	PNH1301	Brazil	SP	ON022619|Brazil|SP|Monkey|2017-04-28
ON022620	2017-12-14	Monkey	PNH2683	Brazil	SP	ON022620|Brazil|SP|Monkey|2017-12-14
ON022621	2017-04-12	Monkey	PNH1262	Brazil	SP	ON022621|Brazil|SP|Monkey|2017-04-12
ON022622	2017-12-07	Monkey	PNH2691	Brazil	SP	ON022622|Brazil|SP|Monkey|2017-12-07
ON022623	2017-12-13	Monkey	PNH2686	Brazil	SP	ON022623|Brazil|SP|Monkey|2017-12-13
ON022624	2017-12-17	Monkey	PNH2688	Brazil	SP	ON022624|Brazil|SP|Monkey|2017-12-17
ON022625	2017-12-19	Monkey	PNH2694	Brazil	SP	ON022625|Brazil|SP|Monkey|2017-12-19
ON022626	2017-11-14	Monkey	PNH2402	Brazil	SP	ON022626|Brazil|SP|Monkey|2017-11-14
ON022627	2017-12-12	Monkey	PNH2702	Brazil	SP	ON022627|Brazil|SP|Monkey|2017-12-12
ON022628	2017-12-12	Monkey	PNH2707	Brazil	SP	ON022628|Brazil|SP|Monkey|2017-12-12
ON022629	2017-12-12	Monkey	PNH2715	Brazil	SP	ON022629|Brazil|SP|Monkey|2017-12-12
ON022630	2017-12-21	Monkey	PNH2725	Brazil	SP	ON022630|Brazil|SP|Monkey|2017-12-21
ON022631	2017-12-21	Monkey	PNH2728	Brazil	SP	ON022631|Brazil|SP|Monkey|2017-12-21
ON022632	2017-12-20	Monkey	PNH2731	Brazil	SP	ON022632|Brazil|SP|Monkey|2017-12-20
ON022633	2017-12-22	Monkey	PNH2743	Brazil	SP	ON022633|Brazil|SP|Monkey|2017-12-22
ON022634	2017-11-07	Monkey	PNH2399	Brazil	SP	ON022634|Brazil|SP|Monkey|2017-11-07
ON022635	2017-07-20	Monkey	PNH1830	Brazil	SP	ON022635|Brazil|SP|Monkey|2017-07-20
ON022636	2017-10-19	Monkey	PNH2636	Brazil	SP	ON022636|Brazil|SP|Monkey|2017-10-19
ON022637	2017-11-14	Monkey	PNH2405	Brazil	SP	ON022637|Brazil|SP|Monkey|2017-11-14
ON022638	2017-10-13	Monkey	PNH2635	Brazil	SP	ON022638|Brazil|SP|Monkey|2017-10-13
ON022639	2017-11-07	Monkey	PNH2632	Brazil	SP	ON022639|Brazil|SP|Monkey|2017-11-07
ON022640	2017-09-21	Monkey	PNH2573	Brazil	SP	ON022640|Brazil|SP|Monkey|2017-09-21
ON022641	2017-09-21	Monkey	PNH2576	Brazil	SP	ON022641|Brazil|SP|Monkey|2017-09-21
ON022642	2017-12-05	Monkey	PNH2514	Brazil	SP	ON022642|Brazil|SP|Monkey|2017-12-05
ON022643	2017-04-25	Monkey	PNH1227	Brazil	SP	ON022643|Brazil|SP|Monkey|2017-04-25
ON022644	2017-10-27	Monkey	PNH2547	Brazil	SP	ON022644|Brazil|SP|Monkey|2017-10-27
ON022645	2017-10-25	Monkey	PNH2550	Brazil	SP	ON022645|Brazil|SP|Monkey|2017-10-25
ON022646	2017-04-25	Monkey	PNH1230	Brazil	SP	ON022646|Brazil|SP|Monkey|2017-04-25
ON022647	2017-04-20	Monkey	PNH1235	Brazil	SP	ON022647|Brazil|SP|Monkey|2017-04-20
ON022648	2017-09-20	Monkey	PNH2030	Brazil	SP	ON022648|Brazil|SP|Monkey|2017-09-20

ON022649	2017-09-25	Monkey	PNH2301		Brazil	SP	ON022649|Brazil|SP|Monkey|2017-09-25
ON022650	2017-11-28	Monkey	PNH2408		Brazil	SP	ON022650|Brazil|SP|Monkey|2017-11-28
ON022651	2017-09-06	Monkey	PNH2570		Brazil	SP	ON022651|Brazil|SP|Monkey|2017-09-06
ON022652	2017-11-30	Monkey	PNH2473		Brazil	SP	ON022652|Brazil|SP|Monkey|2017-11-30
ON022653	2017-12-04	Monkey	PNH2479		Brazil	SP	ON022653|Brazil|SP|Monkey|2017-12-04
ON022654	2017-12-04	Monkey	PNH2482		Brazil	SP	ON022654|Brazil|SP|Monkey|2017-12-04
ON022655	2017-12-05	Monkey	PNH2511		Brazil	SP	ON022655|Brazil|SP|Monkey|2017-12-05
ON022656	2018-02-12	Monkey	PNH3122		Brazil	SP	ON022656|Brazil|SP|Monkey|2018-02-12
ON022657	2018-01-15	Monkey	PNH3123		Brazil	SP	ON022657|Brazil|SP|Monkey|2018-01-15
ON022658	2018-01-29	Monkey	PNH3071		Brazil	SP	ON022658|Brazil|SP|Monkey|2018-01-29
ON022659	2018-02-02	Monkey	PNH3073		Brazil	SP	ON022659|Brazil|SP|Monkey|2018-02-02
ON022660	2018-06-15	Monkey	PNH4225/3		Brazil	SP	ON022660|Brazil|SP|Monkey|2018-06-15
ON022661	2017-03-09	Monkey	PNH642		Brazil	SP	ON022661|Brazil|SP|Monkey|2017-03-09
ON022662	2017-05-05	Monkey	PNH1395		Brazil	SP	ON022662|Brazil|SP|Monkey|2017-05-05
ON022663	2017-09-01	Monkey		1954	Brazil	SP	ON022663|Brazil|SP|Monkey|2017-09-01
ON022664	2017-09-25	Monkey	PNH2302		Brazil	SP	ON022664|Brazil|SP|Monkey|2017-09-25
ON022665	2017-11-06	Monkey	PNH2294		Brazil	SP	ON022665|Brazil|SP|Monkey|2017-11-06
ON022666	2017-12-11	Monkey	PNH2811		Brazil	SP	ON022666|Brazil|SP|Monkey|2017-12-11
ON022667	2017-12-20	Monkey	PNH2799		Brazil	SP	ON022667|Brazil|SP|Monkey|2017-12-20
ON022668	2018-03-20	Monkey	PNH3552		Brazil	SP	ON022668|Brazil|SP|Monkey|2018-03-20
ON022669	2018-03-26	Monkey	PNH3555		Brazil	SP	ON022669|Brazil|SP|Monkey|2018-03-26
ON022670	2018-01-06	Monkey	PNH2890		Brazil	SP	ON022670|Brazil|SP|Monkey|2018-01-06
ON022671	2018-01-06	Monkey	PNH2888		Brazil	SP	ON022671|Brazil|SP|Monkey|2018-01-06
ON022672	2018-01-07	Monkey	PNH2887		Brazil	SP	ON022672|Brazil|SP|Monkey|2018-01-07
ON022673	2018-03-14	Monkey	PNH3557		Brazil	SP	ON022673|Brazil|SP|Monkey|2018-03-14
ON022674	2018-03-19	Monkey	PNH3560		Brazil	SP	ON022674|Brazil|SP|Monkey|2018-03-19
ON022675	2018-03-10	Monkey	PNH3565		Brazil	SP	ON022675|Brazil|SP|Monkey|2018-03-10
ON022676	2019-04-16	Monkey	PNH5475		Brazil	SP	ON022676|Brazil|SP|Monkey|2019-04-16
ON022677	2017-04-10	Monkey	PNH1019		Brazil	SP	ON022677|Brazil|SP|Monkey|2017-04-10
ON022679	2017-03-31	Monkey	PNH1045		Brazil	SP	ON022679|Brazil|SP|Monkey|2017-03-31
ON022680	2017-04-06	Monkey	PNH1041		Brazil	SP	ON022680|Brazil|SP|Monkey|2017-04-06
ON022681	2017-04-10	Monkey	PNH1038		Brazil	SP	ON022681|Brazil|SP|Monkey|2017-04-10
ON022682	2017-03-17	Monkey	PNH733		Brazil	SP	ON022682|Brazil|SP|Monkey|2017-03-17
ON022683	2017-03-17	Monkey	PNH772		Brazil	SP	ON022683|Brazil|SP|Monkey|2017-03-17

ON022684	2017-03-16	Monkey	PNH713	Brazil	SP	ON022684|Brazil|SP|Monkey|2017-03-16
ON022685	2017-03-21	Monkey	PNH702	Brazil	SP	ON022685|Brazil|SP|Monkey|2017-03-21
ON022686	2016-12-09	Monkey	PNH185	Brazil	SP	ON022686|Brazil|SP|Monkey|2016-12-09
ON022687	2017-03-28	Monkey	PNH866	Brazil	SP	ON022687|Brazil|SP|Monkey|2017-03-28
ON022688	2016-11-21	Monkey	PNH149	Brazil	SP	ON022688|Brazil|SP|Monkey|2016-11-21
ON022689	2017-03-23	Monkey	PNH885	Brazil	SP	ON022689|Brazil|SP|Monkey|2017-03-23
ON022690	2017-03-25	Monkey	PNH862	Brazil	SP	ON022690|Brazil|SP|Monkey|2017-03-25
ON022691	2017-03-21	Monkey	PNH872	Brazil	SP	ON022691|Brazil|SP|Monkey|2017-03-21
ON022692	2017-03-29	Monkey	PNH979	Brazil	SP	ON022692|Brazil|SP|Monkey|2017-03-29
ON022693	2017-03-23	Monkey	PNH876	Brazil	SP	ON022693|Brazil|SP|Monkey|2017-03-23
ON022694	2018-03-25	Monkey	PNH3681	Brazil	SP	ON022694|Brazil|SP|Monkey|2018-03-25
ON022695	2018-01-25	Monkey	PNH2998	Brazil	SP	ON022695|Brazil|SP|Monkey|2018-01-25
ON022696	2019-01-15	Monkey	isolada	Brazil	SP	ON022696|Brazil|SP|Monkey|2019-01-15
ON022697	2018-01-03	Monkey	PNH2874	Brazil	SP	ON022697|Brazil|SP|Monkey|2018-01-03
ON022698	2016-11-16	Monkey	PNH72	Brazil	SP	ON022698|Brazil|SP|Monkey|2016-11-16
ON022699	2017-12-27	Monkey	PNH2877	Brazil	SP	ON022699|Brazil|SP|Monkey|2017-12-27
ON022700	2016-11-17	Monkey	PNH96	Brazil	SP	ON022700|Brazil|SP|Monkey|2016-11-17
ON022701	2016-11-17	Monkey	PNH102	Brazil	SP	ON022701|Brazil|SP|Monkey|2016-11-17
ON022702	2016-11-07	Monkey	PNH65	Brazil	SP	ON022702|Brazil|SP|Monkey|2016-11-07
ON022703	2016-11-04	Monkey	PNH69	Brazil	SP	ON022703|Brazil|SP|Monkey|2016-11-04
ON022704	2017-03-31	Monkey	PNH946	Brazil	SP	ON022704|Brazil|SP|Monkey|2017-03-31
ON022705	2017-04-10	Monkey	PNH1258	Brazil	SP	ON022705|Brazil|SP|Monkey|2017-04-10
ON022706	2017-07-27	Monkey	PNH1851	Brazil	SP	ON022706|Brazil|SP|Monkey|2017-07-27
ON022707	2017-12-16	Monkey	PNH2670	Brazil	SP	ON022707|Brazil|SP|Monkey|2017-12-16
ON022708	2017-08-03	Monkey	PNH1869	Brazil	SP	ON022708|Brazil|SP|Monkey|2017-08-03
ON022709	2017-12-18	Monkey	PNH2662	Brazil	SP	ON022709|Brazil|SP|Monkey|2017-12-18
ON022710	2017-12-16	Monkey	PNH2673	Brazil	SP	ON022710|Brazil|SP|Monkey|2017-12-16
ON022711	2017-12-14	Monkey	PNH2676	Brazil	SP	ON022711|Brazil|SP|Monkey|2017-12-14
ON022712	2017-12-13	Monkey	PNH2680	Brazil	SP	ON022712|Brazil|SP|Monkey|2017-12-13
ON022713	2017-11-06	Monkey	PNH2631	Brazil	SP	ON022713|Brazil|SP|Monkey|2017-11-06
ON022714	2017-12-06	Monkey	PNH2485	Brazil	SP	ON022714|Brazil|SP|Monkey|2017-12-06
ON022715	2017-11-15	Monkey	PNH2505	Brazil	SP	ON022715|Brazil|SP|Monkey|2017-11-15
ON022716	2004-11-26	Human	258595_isolado	Brazil		ON022716|Brazil||Human|2004-11-26
ON022717	2000-01-18	Human	188002_isolado	Brazil	SP	ON022717|Brazil|SP|Human|2000-01-18

ON022718	2000-04-27	Human	189642_isolado	Brazil	SP	ON022718|Brazil|SP|Human|2000-04-27
ON022720	2000-01-18	Human	188057_isolado	Brazil	SP	ON022720|Brazil|SP|Human|2000-01-18
ON022723	2017-01-27	Monkey	0441/2017	Brazil	ES	ON022723|Brazil|ES|Monkey|2017-01-27
ON022724	2018-07-02	Human	2501/2018	Brazil	RJ	ON022724|Brazil|RJ|Human|2018-07-02
ON022726	2018-02-20	Human	2538/2018	Brazil	RJ	ON022726|Brazil|RJ|Human|2018-02-20
ON022727	2018-01-26	Human	0869/2018	Brazil	RJ	ON022727|Brazil|RJ|Human|2018-01-26
ON022728	2018-09-02	Human	1778/2018	Brazil	RJ	ON022728|Brazil|RJ|Human|2018-09-02
ON022730	2017-02-14	Human	1344/2017	Brazil	ES	ON022730|Brazil|ES|Human|2017-02-14
ON022731	2017-05-02	Human	1262/2017	Brazil	ES	ON022731|Brazil|ES|Human|2017-05-02
ON022732	2017-02-18	Monkey	1330/2017	Brazil	ES	ON022732|Brazil|ES|Monkey|2017-02-18
ON022733	2018-06-04	Monkey	4838/2018	Brazil	RJ	ON022733|Brazil|RJ|Monkey|2018-06-04
ON022734	2017-06-03	Monkey	2075/2017	Brazil	ES	ON022734|Brazil|ES|Monkey|2017-06-03
ON022735	2017-06-06	Monkey	5884/2017	Brazil	RJ	ON022735|Brazil|RJ|Monkey|2017-06-06
ON022736	2018-01-16	Human	ARB1019	Brazil	SP	ON022736|Brazil|SP|Human|2018-01-16
ON022737	2018-01-19	Human	ARB1029	Brazil	SP	ON022737|Brazil|SP|Human|2018-01-19
ON022738	2018-01-19	Human	ARB1030	Brazil	SP	ON022738|Brazil|SP|Human|2018-01-19
ON022739	2018-01-23	Human	ARB1032	Brazil	SP	ON022739|Brazil|SP|Human|2018-01-23
ON022740	2018-01-30	Human	ARB1045	Brazil	SP	ON022740|Brazil|SP|Human|2018-01-30
ON022741	2018-02-01	Human	ARB1055	Brazil	SP	ON022741|Brazil|SP|Human|2018-02-01
ON022742	2018-01-12	Human	ARB1014	Brazil	SP	ON022742|Brazil|SP|Human|2018-01-12
ON022743	2018-01-16	Human	ARB1020	Brazil	SP	ON022743|Brazil|SP|Human|2018-01-16
ON022744	2018-01-23	Human	ARB1036	Brazil	SP	ON022744|Brazil|SP|Human|2018-01-23
ON022746	2018-XX-XX	Human	ARB1051	Brazil	SP	ON022746|Brazil|SP|Human|2018-XX-XX
ON245434	2020-02-XX	Human		Nigeria		ON245434|Nigeria||Human|2020-02-XX
ON245435	2020-11-XX	Human		Nigeria		ON245435|Nigeria||Human|2020-11-XX
DQ118157	2004-XX-XX			Spain		DQ118157|Spain|2004-XX-XX
GQ379162	2007-10-XX			Peru		GQ379162|Peru|2007-10-XX
GQ379163	2007-10-XX			Peru		GQ379163|Peru|2007-10-XX
JN620362	2010-10-XX			Uganda		JN620362|Uganda|2010-10-XX
JX898868	1995-XX-XX			Senegal		JX898868|Senegal|1995-XX-XX
JX898869	1973-XX-XX			Cote_d_ivoire		JX898869|Cote_d_ivoire|1973-XX-XX
JX898870	1996-XX-XX			Senegal		JX898870|Senegal|1996-XX-XX
JX898871	1995-XX-XX			Senegal		JX898871|Senegal|1995-XX-XX
JX898872	1995-XX-XX			Senegal		JX898872|Senegal|1995-XX-XX

JX898873	2000-XX-XX	Senegal	JX898873|Senegal|2000-XX-XX
JX898874	2000-XX-XX	Senegal	JX898874|Senegal|2000-XX-XX
JX898875	2000-XX-XX	Senegal	JX898875|Senegal|2000-XX-XX
JX898876	2001-XX-XX	Senegal	JX898876|Senegal|2001-XX-XX
JX898877	2005-XX-XX	Senegal	JX898877|Senegal|2005-XX-XX
JX898878	2005-XX-XX	Senegal	JX898878|Senegal|2005-XX-XX
JX898879	2005-XX-XX	Senegal	JX898879|Senegal|2005-XX-XX
JX898880	2005-XX-XX	Senegal	JX898880|Senegal|2005-XX-XX
JX898881	2005-XX-XX	Senegal	JX898881|Senegal|2005-XX-XX
KU921608	2016-03-10	China	KU921608|China|2016-03-10
KU978763	2016-03-09		KU978763||2016-03-09
KU978764	1946-XX-XX	Nigeria	KU978764|Nigeria|1946-XX-XX
KU978765	1941-06-06	Sudan	KU978765|Sudan|1941-06-06
KX010994	1965-08-23	Guinea-bissau	KX010994|Guinea-bissau|1965-08-23
KX010995	2016-03-11	China	KX010995|China|2016-03-11
KX010996	2016-03-17	China	KX010996|China|2016-03-17
KX027336	2016-03-18	China	KX027336|China|2016-03-18
KX268355	2016-03-19	China	KX268355|China|2016-03-19
KX982182	2016-03-11	China	KX982182|China|2016-03-11
KY495641	2016-03-16	Angola	KY495641|Angola|2016-03-16
KY587416	2016-03-11	China	KY587416|China|2016-03-11
KY873606	2016-03-14	China	KY873606|China|2016-03-14
KY873607	2016-03-21	China	KY873607|China|2016-03-21
KY873608	2016-03-23	China	KY873608|China|2016-03-23
MF004382	1999-01-01	Bolivia	MF004382|Bolivia|1999-01-01
MF289572	2017-06-XX	Singapore	MF289572|Singapore|2017-06-XX
MF405338	1927-XX-XX	Ghana	MF405338|Ghana|1927-XX-XX
MF926243	2015-XX-XX	France	MF926243|France|2015-XX-XX
MG589641	2016-03-18	China	MG589641|China|2016-03-18
MG922934	2017-XX-XX	Germany	MG922934|Germany|2017-XX-XX
MH633684	2016-XX-XX	China	MH633684|China|2016-XX-XX
MH633685	2016-XX-XX	China	MH633685|China|2016-XX-XX
MH633686	2016-XX-XX	China	MH633686|China|2016-XX-XX
MH633687	2016-XX-XX	China	MH633687|China|2016-XX-XX

MH633688	2016-XX-XX	China	MH633688|China|2016-XX-XX
MH633689	2016-XX-XX	China	MH633689|China|2016-XX-XX
MH633690	2016-XX-XX	China	MH633690|China|2016-XX-XX
MH633691	2016-XX-XX	China	MH633691|China|2016-XX-XX
MH633692	2016-XX-XX	China	MH633692|China|2016-XX-XX
MK060080	1979-10-30	Senegal	MK060080|Senegal|1979-10-30
MK292067	2018-11-19	Netherlands	MK292067|Netherlands|2018-11-19
MK457701	2018-10-22	Nigeria	MK457701|Nigeria|2018-10-22
MN958078	2018-XX-XX	Nigeria	MN958078|Nigeria|2018-XX-XX
