# Supplementary material for: Yellow Fever Virus Maintained by Sabethes Mosquitoes during the Dry Season in Cerrado, a Semiarid Region of Brazil, in 2021
Source: Viruses. 2023 Mar 15;15(3):757. doi: 10.3390/v15030757 (PMC10058068; doi:10.3390/v15030757)
Supplement: Supplementary file 1 [file viruses-15-00757-s001.zip › Suppl. material S3 2147632.pdf]

In order to reconstruct a Bayesian phylogenetic analysis (Suppl. Fig. 2), we first extracted all the sequences classified within the South American genotype I. We then conducted a local BLAST (Altschul et al., 1990) search of the two recently sequenced genomes against the South American genotype I and from those we selected the 100 sequences with the highest similarity score. This strategy aimed to keep a number of sequences within a computationally tractable range in order to rapidly answer the reviewer.

We explored the temporal signal and the quality of our data set using TempEst v.1.5.3 (Rambaut et al., 2016) and a temporal signal in the final dataset was confirmed (R^2 of 0.58 and correlation coefficient of 0.76). Spatial and temporal patterns of diffusion were estimated using a Bayesian Markov Chain Monte Carlo (MCMC) approach implemented in BEAST v1.10.4 (Suchard et al., 2018) with BEAGLE v3.1.2 (Ayres et al., 2012) to improve computational time.

The general time-reversible model incorporating invariant sites with gamma-distributed rate variation substitution model (GTR+I+G) was used to model nucleotide evolution under the more flexible uncorrelated relaxed molecular clock model with a Bayesian skyline model (BSP; 10 groups) (Gill et al., 2013). Two independent MCMC chains were performed with a length of 100 million generations sampling every 10,000 generations. The two independent runs were merged with Log Combiner v1.10.4 (Suchard et al., 2018) and the convergence of the MCMC chain was assessed using Tracer v1.7.2 (Rambaut et al., 2018). A maximum clade credibility tree (MCC) with annotated branches was then generated in TreeAnnotator v1.10.4, with 20% removed as burn-in (Suchard et al., 2018). The MCC phylogenetic trees were visualized using ggtree R package.

From the Bayesian phylogenetic analysis, we could confirm the patterns observed in our Maximum Likelihood phylogenetic tree (Figure 3 in the paper).

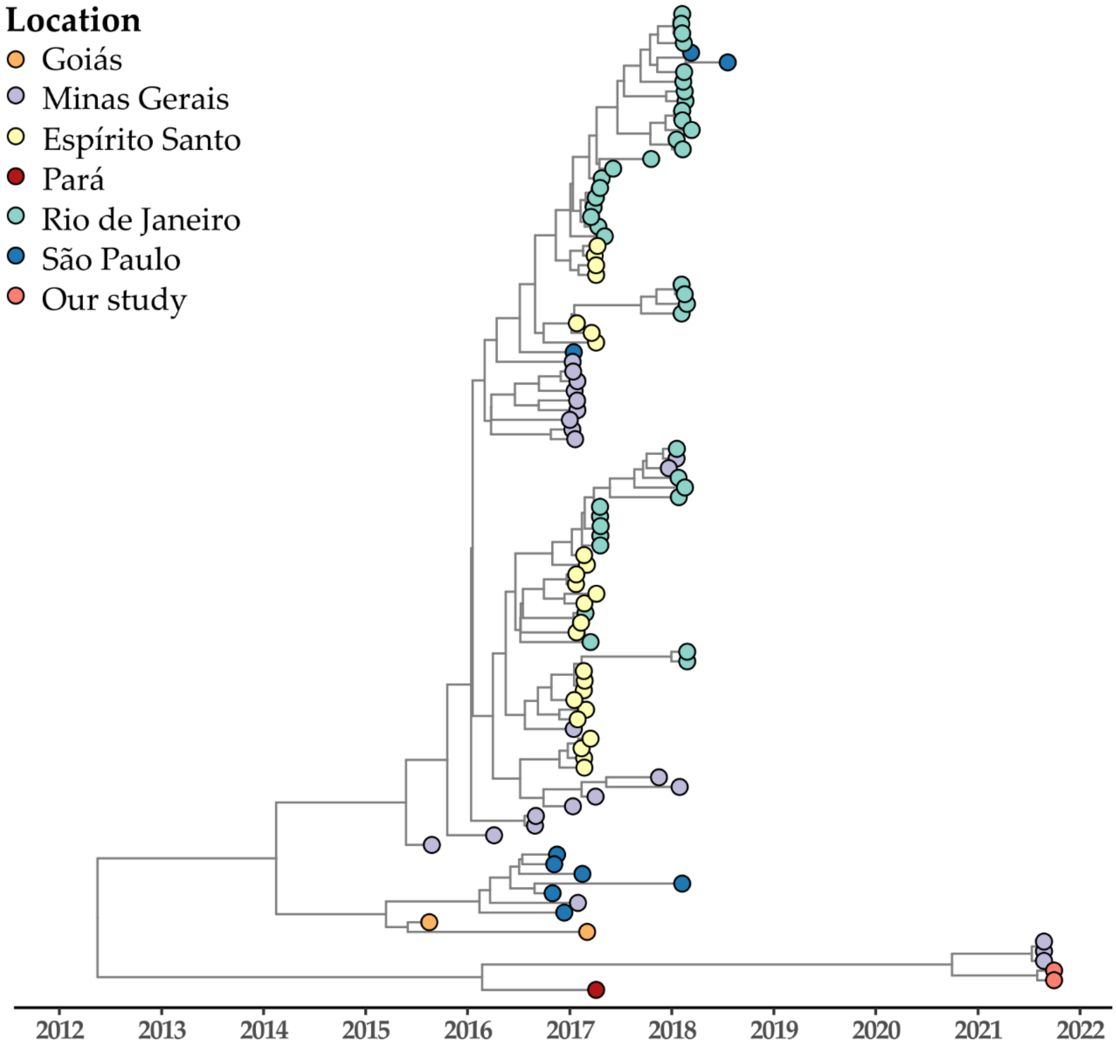

Suppl. Figure S2. Time-scaled phylogenetic tree of 100 complete and near-complete South American genotype I genome sequences and the two new genomes generated in our study sampled in Minas Gerais. Colors represent different sampling locations according to the legend on the left of the tree. Genomes reported in the presented study are colored in red.

Ayres, D.L.; Darling, A.; Zwickl, D.J.; Beerli, P.; Holder, M.T.; Lewis, P.O.; Huelsenbeck, J.P.; Ronquist, F.; Swofford, D.L.; Cummings, M.P. BEAGLE: An Application Programming Interface and High-Performance Computing Library for Statistical Phylogenetics. *Syst. Biol.* **2012**, *61*, 170–173.

Gill, M.S.; Lemey, P.; Faria, N.R.; Rambaut, A.; Shapiro, B.; Suchard, M.A. Improving bayesian population dynamics inference: A coalescent-based model for multiple loci. *Mol Biol Evol.* **2013**, 30, 713–724.

Rambaut, A.; Lam, T.T.; Max Carvalho, L.; Pybus, O.G. Exploring the temporal structure of heterochronous sequences using TempEst (formerly Path-O-Gen). Virus Evol. 2016, 2.

Rambaut, A.; Drummond, A.J.; Xie, D.; Baele, G.; Suchard, M.A. Posterior Summarization in Bayesian Phylogenetics Using Tracer 1.7. *Syst. Biol.* **2018**, 67, 901–904.

Suchard, M.A.; Lemey, P.; Baele, G.; Ayres, D.L.; Drummond, A.J.; Rambaut, A. Bayesian phylogenetic and phylodynamic data integration using BEAST 1.10. *Virus Evol.* **2018**, *4*.
